# Functional roles of Na^+^/K^+^-ATPase in active ammonia excretion and seawater acclimation in the giant mudskipper, *Periophthalmodon schlosseri*

**DOI:** 10.3389/fphys.2014.00158

**Published:** 2014-04-23

**Authors:** Shit F. Chew, Kum C. Hiong, Sock P. Lam, Seow W. Ong, Wei L. Wee, Wai P. Wong, Yuen K. Ip

**Affiliations:** ^1^Natural Sciences and Science Education, National Institute of Education, Nanyang Technological UniversitySingapore, Singapore; ^2^Department of Biological Sciences, National University of SingaporeSingapore, Singapore

**Keywords:** air-breathing fish, ammonia toxicity, gills, ionocytes, nitrogen metabolism, osmoregulation

## Abstract

The giant mudskipper, *Periophthalmodon schlosseri*, is an amphibious fish that builds burrows in the mudflats. It can actively excrete ammonia through its gills, and tolerate high environmental ammonia. This study aimed to examine the effects of seawater (salinity 30; SW) acclimation and/or environmental ammonia exposure on the kinetic properties of Na^+^/K^+^-ATPase (Nka) from, and mRNA expression and protein abundance of *nka*/Nka α–subunit isoforms in, the gills of *P. schlosseri* pre-acclimated to slightly brackish water (salinity 3; SBW). Our results revealed that the Nka from the gills of *P. schlosseri* pre-acclimated to SBW for 2 weeks had substantially higher affinity to (or lower *K*_*m*_ for) K^+^ than NH^+^_4_, and its affinity to NH^+^_4_ decreased significantly after 6-days exposure to 75 mmol l^−1^ NH_4_Cl in SBW. Hence, Nka transported K^+^ selectively to maintain intracellular K^+^ homeostasis, instead of transporting NH^+^_4_ from the blood into ionocytes during active NH^+^_4_ excretion as previously suggested. Two *nka*α isoforms, *nka*α*1* and *nka*α*3*, were cloned and sequenced from the gills of *P. schlosseri*. Their deduced amino acid sequences had K^+^ binding sites identical to that of Nkaα1c from *Anabas testudineus*, indicating that they could effectively differentiate K^+^ from NH^+^_4_. Six days of exposure to 75 mmol l^−1^ NH_4_Cl in SBW, or to SW with or without 50 mmol l^−1^ NH_4_Cl led to significant increases in Nka activities in the gills of *P. schlosseri*. However, a significant increase in the comprehensive Nkaα protein abundance was observed only in the gills of fish exposed to 50 mmol l^−1^ NH_4_Cl in SW. Hence, post-translational modification could be an important activity modulator of branchial Nka in *P. schlosseri*. The fast modulation of Nka activity and concurrent expressions of two branchial *nka*α isoforms could in part contribute to the ability of *P. schlosseri* to survive abrupt transfer between SBW and SW or abrupt exposure to ammonia.

## Introduction

Majority of fully aquatic fishes are ammonotelic, excreting more than 50% of their nitrogenous waste as ammonia-N through their gills. Air-breathing is one of the several adaptive responses utilized by fishes dwelling in habitats with low O_2_ tension (Graham, 1997). With the development of air-breathing abilities, some fishes can emerge from water, make excursions on land, or even burrow into mud during droughts. Air-breathing fishes would experience difficulties in excreting ammonia during emersion or exposure to puddles of water with high concentrations of ammonia. Since ammonia is toxic, air-breathing fishes adopt various strategies to ameliorate ammonia toxicity during emersion or ammonia exposure (Ip et al., 2001a, 2004a,b; Chew et al., 2006; Ip and Chew, 2010; Chew and Ip, 2014). Theoretically, the most effective strategy to defend against ammonia toxicity is to actively excrete ammonia, which would maintain a low internal ammonia concentration and prevent the brain from ammonia intoxication. Indeed, three air-breathing tropical fishes, *Periophthalmodon schlosseri* (Randall et al., 1999; Chew et al., 2003, 2007), *Anabas testudineus* (Tay et al., 2006; Ip et al., 2012a,b; Loong et al., 2012) and *Clarias gariepinus*; (Ip et al., 2004c) are capable of active ammonia excretion through their gills.

The giant mudskipper, *P. schlosseri*, belongs to Class Actinopterygii, Order Perciformes and Family Gobiidae. It is carnivorous and is the only species of mudskippers not found outside the tropics. It inhabits muddy shores in estuaries and in the intertidal zone of rivers, where salinity fluctuates twice daily during high and low tides, in South East Asia. It is an osmoregulator and maintains its plasma osmolality at 260–280 mosmolal in a hypoosmotic or hyperosmotic external medium (Chew and Ip, unpublished results). *Periophthalmodon schlosseri* can survive terrestrial exposure better than other species of mudskippers (Ip et al., 1993; Kok et al., 1998) because it has branched gill filaments with intrafilamentous interlamellar fusions (Low et al., 1988, 1990; Wilson et al., 1999, 2000). The interlamellar fusions form fenestrae which trap water and prevent dehydration when the fish is on land (Low et al., 1988). Naturally, these fusions render aquatic respiration ineffective, but they would facilitate continual ammonia excretion during emersion (Chew et al., 2007). When *P. schlosseri* is exposed to air, ammonia is actively excreted into the small amount of water trapped in the fenestrae, and it builds up quickly therein, reaching very high concentrations (~30 mmol l^−1^; Chew et al., 2007). The ability to actively excrete ammonia against inwardly directed P_NH3_ and NH^+^_4_ concentration gradient also confers *P. schlosseri* the ability to tolerate high concentrations of environmental ammonia (Peng et al., 1998). Unlike many other teleosts which cannot bear 1–5 mmol l^−1^ NH_4_Cl (concentrations which can hardly be regarded as “high environmental ammonia” in the literature) for more than a few hours, *P. schlosseri* can survive in 100 mmol l^−1^ NH_4_Cl in water of salinity 15 for more than a week (Peng et al., 1998). As *P. schlosseri* can actively excrete ammonia, it can maintain low concentrations of ammonia in its plasma when exposed to environmental ammonia, (Peng et al., 1998; Randall et al., 1999) and an unchanged ammonia excretion rate when exposed to alkaline water (Chew et al., 2003).

Active ammonia excretion in *P. schlosseri* can be inhibited by ouabain or amiloride. Thus, Randall et al. (1999) suggested that NH^+^_4_ was transported from the blood into the ionocyte by the basolateral Na^+^/K^+^-ATPase (Nka) with NH^+^_4_ substituting for K^+^, and from the ionocyte to the external medium by the apical Na^+^/H^+^ (NH^+^_4_) exchanger. However, if NH^+^_4_ were to be actively transported into ionocytes through basolateral Nka (Weihrauch et al., 2009; Hwang et al., 2011), it must have comparable *K*_*m*_ values and be able to effectively replace K^+^ to activate Nka. In that case, it would be difficult for the ionocyte to maintain high intracellular [K^+^] to uphold intracellular K^+^ homeostasis and low [NH^+^_4_] to minimize ammonia cytotoxicity. The fundamental principle is that ammonia is cytotoxic and therefore needs to be excreted instead of allowing its intracellular concentration to rise (Ip and Chew, 2010). More importantly, active excretion of NH^+^_4_ naturally involves the transport of NH^+^_4_ against an electrochemical gradient across the apical, and not the basolateral, membrane of ionocytes. With active excretion of NH^+^_4_ to the external medium through the apical membrane, the intracellular ammonia concentration would logically be maintained at low and sub-toxic levels. Moreover, the basolateral membrane generally has an electrical potential of ~75 mV (inside negative; Wright, 1991); hence unlike K^+^ which has a very high intracellular concentration (~120 mmol l^−1^), NH^+^_4_ does not need to be actively transported across the basolateral membrane through Nka.

The climbing perch, *A. testudineus*, is an obligate air-breather and a euryhaline freshwater teleost capable of progressive acclimation from fresh water to seawater (Chang et al., 2007), migration on land, and active excretion of ammonia during emersion or ammonia exposure (Tay et al., 2006). Exposure of *A. testudineus* to ammonia in fresh water results in a significant increase in the mRNA expression and protein abundance of Na^+^:K^+^:2Cl^−^ cotransporter 1a (Nkcc1a) in its gills (Loong et al., 2012). Hence, it is probable that NH^+^_4_ can enter ionocytes through the basolateral Nkcc1a, in substitution of K^+^, before being actively transported across the apical membrane (Loong et al., 2012). Since increased activity of Nkcc1a would lead to an increased influx of Na^+^ and NH^+^_4_ into ionocytes, an up-regulation of branchial Nka activity and an increase in its selectivity for K^+^ over NH^+^_4_ are necessary to maintain the electrochemical potential gradients of Na^+^ and K^+^ during active ammonia excretion (Ip et al., 2012a). NKA/Nka coordinates the active transport of 3 Na^+^ out of and 2 K^+^ into, the cell, fuelled by the hydrolysis of ATP. It contains 2 major subunits, α and β, and functions as an αβ heterodimer. The α-subunit is a large (110–120 kDa) protein that contains all the functional sites and is responsible for the catalytic functioning of the enzyme (Blanco and Mercer, 1998). Four isoforms of the *NKA* α-subunit (α*1*, α*2*, α*3*, α*4*) have been identified in mammals (Blanco and Mercer, 1998). For *A. testudineus*, 3 *nka*α isoforms (α*1a*, α*1b*, and α*1c*) have been cloned and sequenced from its gills recently (Ip et al., 2012a). The mRNA expression of *nka*α*1a* is down-regulated in the gills of *A. testudineus* acclimated to seawater, indicating that it is a freshwater-isoform involving in branchial Na^+^ absorption in a hypoosmotic environment (Ip et al., 2012a). By contrast, seawater acclimation leads to an up-regulation of the mRNA expression of *nka*α*1b* and to a lesser extent *nka*α*1c*, indicating that they are the seawater-isoforms essential for ion secretion in a hyperosmotic environment. More importantly, exposure of *A. testudineus* to ammonia in fresh water leads to a significant increase in the mRNA expression of *nka*α*1c*, indicating that it plays an important role in active ammonia excretion (Ip et al., 2012a). Since there is a decrease in the effectiveness of NH^+^_4_ to substitute for K^+^ in the activation of Nka from the gills of *A. testudineus* exposed to ammonia as compared to the freshwater control (Ip et al., 2012a), the up-regulation of *nka* α*1c* expression probably serves to remove excess Na^+^ from, and to transport K^+^ in preference to NH^+^_4_ into, ionocytes. Similarly, it is probable that the main function of branchial Nka in active NH^+^_4_ excretion in *P. schlosseri* is to maintain intracellular Na^+^ and K^+^ homeostasis, instead of transporting NH^+^_4_ directly into ionocytes as previously proposed (Randall et al., 1999; Weihrauch et al., 2009; Hwang et al., 2011).

Therefore, the first objective of this study was to determine the kinetic properties of Nka from the gills of *P. schlosseri* pre-acclimated to slightly brackish water (salinity 3; SBW) for 2 weeks. We hypothesized that NH^+^_4_ could not effectively substitute for K^+^ to induce Nka activity and therefore the *K*_*m*_ for NH^+^_4_ would be substantially greater than that for K^+^. The second objective was to examine the effects of 6-days exposure to ammonia (75 mmol l^−1^ NH_4_Cl) in SBW, seawater (salinity 30; SW), or ammonia (50 mmol l^−1^ NH_4_Cl) in SW on the *K*_*m*_ and the *V*_max_ of branchial Nka. The hypothesis tested was that kinetic properties of branchial Nka from *P. schlosseri* would be altered not only by seawater acclimation but also by exposure to ammonia in SBW or SW. The third objective was to clone and sequence *nka*α isoforms from the gills of *P. schlosseri*, so as to characterize the Na^+^ and K^+^ binding sites of the deduced Nkaα sequences and to compare their K^+^ binding sites with those of the 3 Nkaα isoforms from *A. testudineus*. Since NKA/Nka activity is known to be regulated through transcription, translation and/or covalent modification, the fourth objective was to determine the effects of 6-days exposure to ammonia (75 mmol l^−1^ NH_4_Cl) in SBW, SW, or ammonia (50 mmol l^−1^ NH_4_Cl) in SW on the mRNA expression of *nka*α isoforms and the comprehensive protein abundance of Nkaα in the gills of *P. schlosseri* pre-acclimated to SBW. Being a euryhaline brackish water fish, *P. schlosseri* can survive abrupt transfer between SBW (or water of salinity as low as 1) and SW without mortality. Hence, we hypothesized that, unlike *A. testudineus* (Ip et al., 2012a), there might not be a clear delineation of freshwater- and seawater-types of *nka*α isoforms in the gills of *P. schlosseri*, and the up-regulation of branchial Nka activity during exposure to SW and/or ammonia might not result from large increases in mRNA and/or protein expression, which are relatively slow processes.

Two different types of abbreviations of genes/proteins have been adopted in this report, as the standard abbreviations of genes/proteins of fishes (http://zfin.org/cgi-bin/webdriver?MIval=aa-ZDB_home.apg) are different from those of human/ non-human primates (http://www.genenames.org). For fishes, gene symbols are italicized, all in lower case, and protein designations are the same as the gene symbol, but not italicized with the first letter in upper case. The advantage of using two types of abbreviations appropriately is that it would allow immediate interpretation of the affiliation between the abbreviated gene/protein and fishes or human/non-human primates.

## Materials and methods

### Fish

Specimens of *P. schlosseri* (90–100 g body mass) were purchased from fishermen at Benut, Malaysia, imported to Singapore, and transferred to the National Institute of Education, Nanyang Technological University. The lowest salinity during low tides and the highest salinity during high tides recorded in the natural habitat of *P. schlosseri* at Benut, Malaysia were salinity 3 (pH 6.8–7.4) and salinity 30 (pH 7.8–8.3), respectively. Procedures adopted in this study were approved by the Institutional Animal Care and Use Committee (IACUC) of the Nanyang Technological University (ARF SBS/NIE-A-0122 AZ).

No attempt was made to separate the sexes. Fish were maintained in SBW at salinity 3 in individual plastic aquaria (L29 × W19 × H17.5 cm) at 25–27°C under a 12 h light:12 h dark regime. Salinity was monitored using a YSI Model 30/10 FT salinometer (Yellow Springs Instrument Co. Inc, Ohio, USA). No aeration was provided because *P. schlosseri* is an obligate air-breather. Since specimens of *P. schlosseri* were obtained from the estuaries where salinity fluctuated twice daily during high and low tides, they might have high branchial *nka*/Nka expression. Therefore, fish were pre-acclimated to SBW for 2 weeks before being exposed to experimental conditions. SBW had an osmolality of ~80 mosmolal, and was hypoosmotic to the plasma osmolality (260–280 mosmolal) of *P. schlosseri*. Hence, similar to freshwater fishes, *P. schlosseri* would have to undergo hyperosmotic regulation to prevent water influx and ion depletion during long-term exposure to SBW. The assumption was that 2 weeks of pre-acclimation in a hypoosmotic medium would be adequate to obtain the baseline expression levels of *nka*/Nka in the gills of *P. schlosseri*. During the pre-acclimation period, *P. schlosseri* was fed fish meat and water was changed daily.

### Experimental conditions and collection of samples

After pre-acclimation to SBW for 2 weeks, fish were divided into 3 groups (total *N* = 24). In the first group of fish, 4 fish were placed in SBW (control) while the other 4 fish were immersed in 20 volumes (v/w) of SBW (pH 6.8–7.0) containing 75 mmol l^−1^ NH_4_Cl (pH 7.0) for 6 days. For the second group of fish, again 4 fish were placed in SBW (control) while the other 4 fish were immersed in 20 volumes (v/w) of SW (pH 8.2) for 6 days. Salinity was monitored as mentioned above. SW was made from Red Sea salt (Houston, TX, USA) and aerated for 24 h in order to obtain a stabilized pH of 8.2 before usage. In order to imitate the physiological condition in the natural habitat of *P. schlosseri*, no attempt was made to adjust the pH of SW. For the third group of fish, 4 fish were immersed in SW (control) while another 4 fish were immersed in 20 volumes (v/w) of SW containing 50 mmol l^−1^ NH_4_Cl (pH 8.0) for 6 days. Preliminary studies revealed that while *P. schlosseri* could survive in 75 mmol l^−1^ NH_4_Cl at pH 7.0 in SBW for an extended period (>2 weeks), it would succumb after 2- to 3-days exposure to 75 mmol l^−1^ NH_4_Cl at pH 8.0 in SW. Of note, the ratio of NH_3_ to NH^+^_4_ at pH 8.0 in SW would be greater than that at pH 7.0 in SBW. As NH_3_ is the predominant species of ammonia that permeate biomembranes, ammonia toxicity increases with increasing environmental pH. Therefore, the concentration of NH_4_Cl was lowered to a sub-lethal concentration of 50 mmol l^−1^ NH_4_Cl (pH 8.0) in SW.

Fish were fed with fish meat during the experimental period, and water was changed daily. No mortality was recorded for fish kept in all conditions. After 6 days, fish were killed with an overdose of neutralized MS-222 (0.2%) followed with a strong blow to the head. Gills were sampled and suspended in 1 ml of solution containing 0.1 mol l^−1^ imidazole-HCl (pH 7.2), 0.3 mol l^−1^ sucrose, 0.02 mol l^−1^ EDTA following the method of Zaugg (1982) for the Nka activity assays and western blotting studies. Tissues collected from all four gill arches constituted one sample. Samples were frozen in liquid nitrogen and stored at −80°C until analyses.

Gill filaments needed for molecular studies were collected from a separate group of fish (total *N* = 40). Fish that have been pre-acclimated to SBW for 2 weeks served as controls (*N* = 4). After 2 weeks of pre-acclimation to SBW, a group of experimental fish (*N* = 4 for each time point) was immersed in 20 volumes (v/w) of SBW containing 75 mmol l^−1^ NH_4_Cl (pH 7.0) for 1, 2, or 6 days with daily changes of NH_4_Cl solution. A second group of experimental fish (*N* = 4 for each time point) was immersed in 20 volumes (v/w) of SW (pH 8.2) for 1, 2, or 6 days with daily changes of SW. A third group of experimental fish (*N* = 4 for each time point) was immersed in 20 volumes (v/w) of SW containing 50 mmol l^−1^ NH_4_Cl (pH 8.0) for 1, 2, or 6 days with daily changes of NH_4_Cl solution. Fish were fed fish meat daily during the experimental period. Gill filaments from both the control and experimental fish were excised, frozen in liquid N_2_, and stored at −80°C until analyses.

### Determination of Nka activity

The gill sample collected by the method of Zaugg (1982) was thawed on ice and homogenized for 2 s at 7000 rpm. The homogenate was then centrifuged at 2000 × *g* for 7 min at 4°C to obtain the pellet. The pellet was re-suspended in 1 ml of homogenizing buffer containing 0.1 mol l^−1^ imidazole-HCl (pH 7.2), 0.3 mol l^−1^ sucrose, and 1 g l^−1^ of sodium deoxycholate (without EDTA, which interfered with the subsequent phosphate analysis), and homogenized twice at 13,500 rpm for 10 s each with an interval of 10 s. The homogenized sample was centrifuged for 6 min at 2000 × *g* and 4°C. The supernatant obtained was assayed for Nka activity on the same day. The protein content of the sample was determined according to the method of Bradford (1976). The quantities of protein used per assay were approximately 0.23, 0.1, and 0.2 mg for samples from SBW control, seawater-acclimated and ammonia-exposed fish, respectively.

The optimized reaction mixture for the determination of Nka activity (*V*_sat_) contained 0.05 ml sample, 30 mmol l^−1^ imidazole-HCl buffer (pH 7.2), 120 mmol l^−1^ NaCl, 20 mmol l^−1^ KCl, 5 mmol l^−1^ MgCl_2_ and 3.5 mmol l^−1^ ATP, with or without 2 mmol l^−1^ ouabain in a total volume of 1 ml. The compositions of Na^+^ and K^+^ (or NH^+^_4_ in replacement of K^+^) were varied in order to obtained the *K*_*m*_ for various substrates (Na^+^, K^+^, and NH^+^_4_) and the corresponding *V*_max_. The reaction mixture without ATP was pre-incubated at 25°C for 10 min and the reaction was initiated by the addition of 0.05 ml of ATP. After 40 min of incubation at 25°C, the reaction was terminated by the addition of 0.05 ml of ice-cold 100% trichloroacetic acid. The mixture was centrifuged at 12,000 × *g* for 2 min at 4°C. Preliminary results indicate that the Nka activity increased linearly with time up to at least 45 min under all conditions. The amount of inorganic phosphate (Pi) released from ATP during the incubation period represented the activity of Nka. An aliquot (0.4 ml) of the supernatant was diluted with 4 volumes of 0.1 mol l^−1^ sodium acetate for Pi assay. To this diluted aliquot, 0.2 ml of 1% ascorbic acid and 0.2 ml of 1% ammonium molybdate in 0.05 mol l^−1^ H_2_SO_4_ were added. The solution was incubated for 30 min at 30°C and the absorbance was determined at 700 nm using a Shimadzu (Kyoto, Japan) UV160 UV-VIS spectrophotometer, and the Pi concentration calculated with reference to a standard made from K_2_HPO_4_ and assayed in the presence of trichloroacetic acid and sodium acetate. The Na^+^/K^+^-ATPase or Na^+^/NH^+^_4_-ATPase activity was calculated as a difference of activities assayed in the presence and absence of ouabain. The activity of Nka is expressed as μmol Pi released min^−1^ mg^−1^ protein.

To obtain *K*_*m*_ and *V*_max_ for Na^+^, effects of different concentrations of Na^+^ (5, 10, 20, and 120 mmol l^−1^) on Nka activity were determined in the presence of 20 mmol l^−1^ K^+^ for all samples. To obtain *K*_*m*_ and *V*_max_ for K^+^ or NH^+^_4_, effects of varying K^+^ or NH^+^_4_ concentrations (1, 2.5, 5, and 20 mmol l^−1^) on the Na^+^/K^+^-ATPase or Na^+^/NH^+^_4_-ATPase activity were determined at 120 mmol l^−1^ Na^+^. The *K*_*m*_ and *V*_max_ values were obtained from the Lineweaver-Burk plot. Since Lineweaver-Burk plots are very susceptible to biases at low and high substrate concentrations, results were further analyzed by the Woolf–Augustinsson plot, which also produced comparable *K*_*m*_ and *V*_max_ values, and confirmed the differences observed between experimental conditions. Since three *V*_max_ values were generated for each samples based on varied Na^+^, K^+^, or NH^+^_4_ concentrations, and since they were generally in agreement with the *V*_sat_ value obtained under optimized assay conditions, *V*_sat_ values were reported instead of *V*_max_.

### Total RNA extraction and cDNA synthesis

Total RNA was extracted from the gill samples collected using Tri Reagent™ (Sigma-Aldrich Co., St. Louis, MO, USA) and further purified using the RNeasy Plus Mini Kit (Qiagen GmbH, Hilden, Germany). The RNA obtained was quantified using a NanoDrop ND-1000 spectrophotometer (Nanodrop Technologies Inc., Wilmington, DE, USA) and its integrity was verified by electrophoresis. The total RNA (1 μg) isolated was reverse transcribed into first strand cDNA using oligo (dT)_18_ primers and the RevertAid™ First Strand cDNA synthesis kit (Thermo Fisher Scientific Inc. Waltham, MA, USA).

### Polymerase chain reaction (PCR), cloning, and RACE-PCR

The partial *nka* α-subunit sequences were obtained using primers (Forward: 5′-CACTTCATCCACATCATCAC-3′ and Reverse: 5′-ATGGCGGGAACCATGTC-3′) designed according to conserved regions of *Anguilla anguilla nka*α (X76108), *Fundulus heteroclitus nka*α*1* (AY057072), *Oncorhynchus mykiss nka*α*1a* (AY319391), *Oreochromis mossambicus nka*α*1* (TMU82549), *F. heteroclitus nka*α*2* (AY057073), *O. mykiss nka*α*2* (NM_001124458), *O. mykiss nka*α*3* (NM_001124630) and *O. mossambicus nka*α*3* (AF109409). Notably, using the same pair of primers, 3 *nka*α*1* isoforms (α*1a*, α*1b*, and α*1c*) have been cloned from the gills of *A. testudineus* (Ip et al., 2012a), and 3 *nka*α isoforms (α*1*, α*2*, and α*3*) have been cloned from the gills of the euryhaline freshwater stingray, *Himantura signifer* (Ip et al., 2013), and the brain of the African lungfish, *Protopterus annectens* (Hiong et al., 2014). In addition, this pair of primers was able to amplify *nka*α*1*, *nka*α*3a* and *nka*α*3b* from the brain of the swamp eel, *Monopterus albus* (Chen et al., 2013).

The PCR was performed in a 9902 Veriti 96-well thermal cycler (Applied Biosystems, Carlsbad, CA, USA) using DreamTaq™ polymerase (Thermo Fisher Scientific Inc.). The cycling conditions were 94°C (3 min), followed by 35 cycles at 94°C (30 s), 50°C (30 s), and 72°C (2 min), and one cycle of final extension at 72°C (7 min). The PCR products obtained were separated by electrophoresis in 1% agarose gel. Bands of the estimated size (1500–1600 bp) were extracted from the gels using Promega Wizard SV gel and PCR cleanup system (Promega Corporation, Madison, WI, USA). The PCR products were cloned into pGEM^®^-T Easy vector (Promega Corporation). The ligated vector was transformed into JM109 competent cells and plated onto Luria-Bertani (LB) agar with 100 μg ml^−1^ ampicillin, 50 μg ml^−1^ X-gal and 0.5 mmol l^−1^ IPTG. Selected white colonies were grown overnight in LB with ampicillin. The plasmids were extracted using the resin-based plasmid miniprep kit (Axygen Biosciences, Union city, CA, USA).

The plasmids were sequenced in both directions using the BigDye^®^ Terminator v3.1 Cycle Sequencing Kit (Applied Biosystems) with a 3130XL Genetic Analyzer (Applied Biosystems). The sequence data generated was viewed and analyzed using BioEdit version 7.0.9 (Hall, 1999). Analysis of multiple clones of *nka*α fragment with the nucleotide database of National Center for Biotechnology Information (http://blast.ncbi.nlm.nih.gov/Blast.cgi) revealed the presence of two *nka* α-subunit isoforms in the gills of *P. schlosseri*.

The complete cDNA sequences of the two *nka* α-subunit isoforms were obtained using SMART™ RACE cDNA amplification kit (Clontech Laboratories, Mountain View, CA, USA) and 5′ and 3′ RACE primers (Table 1) designed. The cDNA sequences of *nka* α-subunit isoforms obtained were assembled and analysed using BioEdit and the complete sequences were deposited into GenBank.

**Table 1:** **Primers used for RACE and quantitative real-time PCR (qPCR) of Na^+^/K^+^-ATPase (nka) α-subunit isoforms from gills of *Periophthalmodon schlosseri***.

**Gene**	**Primer type**		**Primer sequence**
*nka*α*1*	RACE-PCR	5′-RACE	5′-TCCAACGAAGCACAGGTTCTCCGTGG-3′
		3′-RACE	5′-GTGTTCCTGGCTGACCAAAGCAATGTGC-3′
	qPCR	Forward	5′-TGCTACTCTGGCCTCTAGT-3′
		Reverse	5′-CCATATCCGAGGATGAGTGA-3′
*nka*α*3*	RACE-PCR	5′-RACE	5′-GCCAAGTCACCGACGACTTATCAAACGAAG-3′
		3′-RACE	5′-GCTGTCCTGCGGTTCGGTCAGACA-3′
	qPCR	Forward	5′-AGAGTCGGAGCCTCAGAA-3′
		Reverse	5′-GCAGATCACGATTCCACG-3′

### Deduced amino acid sequence and phylogenetic analysis

The amino acid sequences of the two Nka α-subunits were translated from the nucleotide sequences of *nka* α-subunits using ExPASy Proteomic server (http://web.expasy.org/translate/) (Gasteiger et al., 2003). The transmembrane domains of the translated amino acid sequences of both Nka α-subunit isoforms were identified using MEMSAT3 & MEMSAT-SVM provided by PSIPRED protein structure prediction server (http://bioinf.cs.ucl.ac.uk/psipred/) (McGuffin et al., 2000).

The identity of amino acid sequences of both Nkaα isoforms from *P. schlosseri* was confirmed by comparing with various Nkaα sequences for other animals species available in the Genbank and obtaining percentage similarities based on ClustalX2 and Bioedit. Furthermore, a Phylip analysis was performed using the neighbor-joining method (NEIGHBOR) in PHYLIP phylogeny package (version 3.67), with the inclusion of 100 bootstraps (Felsentein, 1989), to generate a phenogram of amino acid sequence similarities to confirm the identities of the deduced amino acid sequences of Nkaα isoforms from the gills of *P. schlosseri*. The phylogenetic tree were generated with CONSENSE using 50% majority rule and plotted using the TREEVIEW program version 1.6.6. (http://taxonomy.zoology.gla.ac.uk/rod/treeview.html) (Page, 1996).

Amino acid sequences of Nkaα from various animals were obtained from Genbank with the following accession numbers: *A. testudineus* Nkaα1a (AFK29492.1), *A. testudineus* Nkaα1b (AFK29493.1), *A. testudineus* Nkaα1c (AFK29494.1), *A. anguilla* Nkaα1 (CAA53714.1), *Danio rerio* Nkaα1 (NP_571761.1), *D. rerio* Nkaα1a.2 (NP_571762.1), *D. rerio* Nkaα1a.3 (NP_571763.1), *D. rerio* Nkaα1a.4 (NP_571764.1), *D. rerio* Nkaα1a.5, *D. rerio* Nkaα1b (NP_571765.1), *D. rerio* Nkaα2 (NP_571758.1), *Salmo salar* Nkaα1 (ACN10460.1), *O. mykiss* Nkaα1a (NP_001117933.1), *O. mykiss* Nkaα1b (NP_001117932.1), *O. mykiss* Nkaα1c (NP_001117931.1), *Oncorhynchus masou* Nkaα1a (BAJ13363.1), *O. masou* Nkaα1b (BAJ13362.1), *O. mykiss* Nkaα2 (NP_001117930.1), *O. mykiss* Nkaα3 (NP_001118102.1), *F. heteroclitus* Nkaα1 (AAL18002.1), *F. heteroclitus* Nkaα2 (AAL18003.1), *Sarotherodon melanotheron* Nkaα1 (ADB03120.1), *O. mossambicus* Nkaα1 (AAD11455.2), *O. mossambicus* Nkaα3 (AAF75108.1), *Chanos chanos* Nkaα1 (ABF58911.1), *Carassius auratus* Nkaα3 (BAB60722.1), *Trematomus bernacchii* Nkaα3 (AAY30258.1), and *Saccoglossus kowalevskii* Nkaα1 (XP_002737354.1; as the outgroup).

### Quantitative real-time PCR (qPCR)

There are two types of quantification methods for qPCR (Wong and Medrano, 2005). In absolute quantification, the precise amount of the template used for the curve is known with expression levels being expressed in absolute numbers of copies. In relative quantification, the template of interest is known to be present in the sample, but its absolute amount is uncertain. However, fold change does not allow the interpretation of which isoform being the predominant one being expressed in a certain condition. Since it is essential to compare the mRNA expression of *nka*α*1* and *nka*α*3* in the gills of *P. schlosseri*, the method of absolute quantification with reference to a standard curve was adopted in this study.

RNA (2 μg) from gill samples of *P. schlosseri* were extracted as mentioned above and reverse-transcribed using random hexamer primers with RevertAid™ first strand cDNA synthesis kit (Thermo Fisher Scientific Inc.). qPCR was performed in triplicates using a StepOnePlus™ Real-Time PCR System (Applied Biosystems). The mRNA expression of the two *nka* α-subunits in the gills of *P. schlosseri* was determined using specific qPCR primers (Table 1).

In order to determine the absolute quantity of the each of the *nka α*-subunit transcripts in a qPCR reaction, efforts were made to produce a pure amplicon (standard) of a defined region of *nka* α-subunits cDNA from the gills of *P. schlosseri* following the method of Gerwick et al. (2007). PCR was performed with the qPCR primers (Table 1) and cDNA as a template in a final volume of 25 μl with the following cycling conditions: initial denaturation 95°C for 3 min, followed by 35 cycles of 95°C for 30 s, 60°C for 30 s and 72°C for 30 s and 1 cycle of final extension of 72°C for 10 min. The PCR product was separated in a 2% agarose gel then excised and purified using Promega Wizard SV gel and PCR cleanup system. The *nka* α-subunit nucleotide fragment in the purified product was cloned using pGEM-T Easy vector. The presence of the insert in the recombinant clones was confirmed by sequencing. The cloned circular plasmid was quantified using a spectrophotometer.

The standard cDNA (template) was serially diluted (from 10^6^ to 10^2^ specific copies per 2 μl). The PCR reactions contained 5 μl of 2× Fast SYBR^®^ Green Master Mix (Applied Biosystems), 0.3 μmol l^−1^ of forward and reverse primers each (Table 1) and 1 ng of sample cDNA or various quantities of standard in a total volume of 10 μl. Cycling conditions were 95°C for 20 s (1 cycle), followed by 40 cycles of 95°C for 3 s, and 60°C for 30 s. Data (Ct values) were collected at each elongation step. A melt curve analysis was performed after each run by increasing the temperature from 60 to 95°C in 0.3°C increments to confirm the presence of a single product only. The PCR products obtained were also separated in a 2% agarose gel to verify the presence of a single band. A standard curve was obtained from plotting threshold cycle (*C*_*t*_) on the Y-axis and the natural log of concentration (copies μl^−1^) on the X-axis. The *C*_*t*_ slope, PCR efficiency, Y-intercept and correlation coefficient (*r*^2^) were calculated using the default setting of StepOne™ Software v2.1. Diluted standards were stored at −20°C. The PCR amplification efficiencies for the *nka*α*1* and *nka*α*3* were 97.8 and 93.7%, respectively. The quantity of transcript in a sample was determined from the linear regression line derived from the standard curve and expressed as copy number per ng cDNA (Gerwick et al., 2007).

The specificity of each pair of qPCR primers was verified by PCR using two different plasmid clones containing fragments of *nka*α*1* and *nka*α*2* as templates. The identities of these plasmid clones had been verified through cloning and sequencing previously (see section above). The specificity of each pair of primers was demonstrated by the presence of a single band using the plasmid clones of the targeted *nka*α isoform as the template and the absence of detectable band using the plasmid clones of the other isoform. Furthermore, for each pair of primers, the *C*_*t*_ value obtained using plasmid clones of the targeted *nka*α fell between 16 and 20, but no detectable *C*_*t*_ values (i.e., undetermined) were obtained using the other plasmid clones.

### SDS-page and western blotting

The gill filaments were homogenized three times in five volumes (w/v) of ice cold buffer containing 50 mmol l^−1^ Tris HCl, (pH 7.4), 1 mmol l^−1^ EDTA, 150 mmol l^−1^ NaCl, 1 mmol l^−1^ NaF, 1 mmol l^−1^ Na_3_VO_4_, 1% NP-40, 1% sodium deoxycholate, 1 mmol l^−1^ PMSF, and 1× HALT protease inhibitor cocktail (Thermo Fisher Scientific Inc.) at 24,000 rpm for 20 s each with 10 s intervals using the Polytron PT 1300D homogenizer (Kinematica AG, Lucerne, Switzerland). The homogenate was centrifuged at 10,000 × *g* for 20 min at 4°C. The protein concentration in the supernatant obtained was determined according to the method of Bradford (1976) and adjusted to 2 μg μl^−1^ with Laemmli buffer (Laemmli, 1970). Samples were heated at 70°C for 15 min, and then kept at −80°C until analysis.

Proteins were separated by SDS-PAGE (8% acrylamide for resolving gel, 4% acrylamide for stacking gel) under conditions as described by Laemmli (1970) using a vertical mini-slab apparatus (Bio-Rad Laboratories, Hercules, CA, USA). Proteins were then electrophoretically transferred onto PVDF membranes using a transfer apparatus (Bio-Rad Laboratories). After transfer, membranes were blocked with 10% skim milk in TTBS (0.05% Tween 20 in Tris-buffered saline: 20 mmol l^−1^ Tris-HCl; 500 mmol l^−1^ NaCl, pH 7.6) for 1 h before being incubated overnight at 4°C with NKA antibody (1:800 dilution) or pan-actin antibody (1:5000 dilution). The NKA antibody, α5, was developed by Douglas M. Farmbrough (Johns Hopkins University, MD, USA) under the auspices of NICHD and maintained by The University of Iowa, Department of Biological Sciences, Iowa City, IA52242, USA. α5 is known to react comprehensively with Nka α-subunit isoforms in fish (Wilson et al., 2007; Ip et al., 2012a). Both α5 and pan-actin antibodies were diluted in 1% BSA in TTBS. The membranes were then incubated in alkaline phosphatase-conjugated secondary antibodies (anti-mouse or anti-goat; 1:10,000 dilutions) for 1 h, rinsed and then incubated for 5 min in a solution of 5-bromo-4-chloro-3-indolyl phosphate *p*-toluidine salt and nitro-blue tetrazolium chloride (Invitrogen, Carlsbad, CA, USA) for color development. The blots were scanned using CanonScan 4400F flatbed scanner in TIFF format at 300 dpi resolution. Densitometric quantification of band intensities were performed using ImageJ (version 1.40, NIH), calibrated with a calibrated 37 step reflection scanner scale (1″ × 8″; Stouffer #R3705-1C). Results were presented as relative protein abundance of Nka normalized with actin.

### Statistical analysis

**Figure 1:**
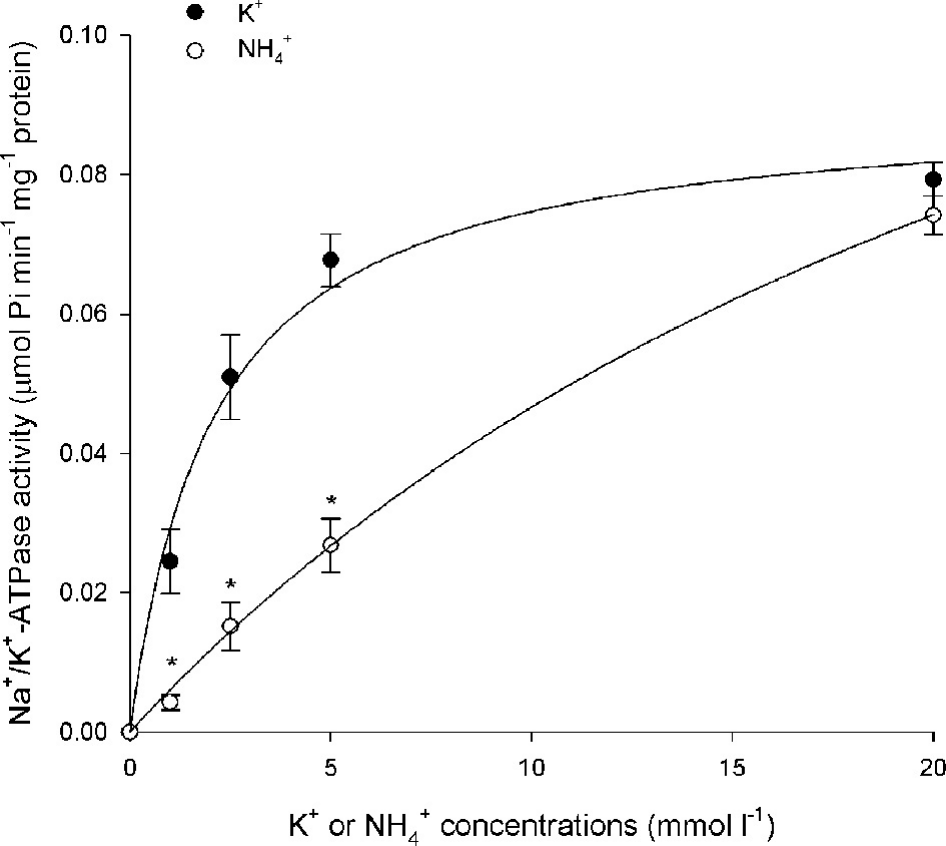
**Effects of varying K^+^ or NH^+^_4_ concentrations on branchial Na^+^/K^+^-ATPase (Nka) activity.** Specific activity (μmol Pi min^−1^ mg^−1^ protein) of Nka from the gills of *Periophthalmodon schlosseri* pre- acclimated to slightly brackish water (SBW; salinity 3) for 2 weeks (control) with varying concentrations of K^+^ or NH^+^_4_. Results represent mean ± s.e.m. (*N* = 4). ^*^Significant difference from the corresponding K^+^-induced specific activity (*P* < 0.05).

**Figure 2:**
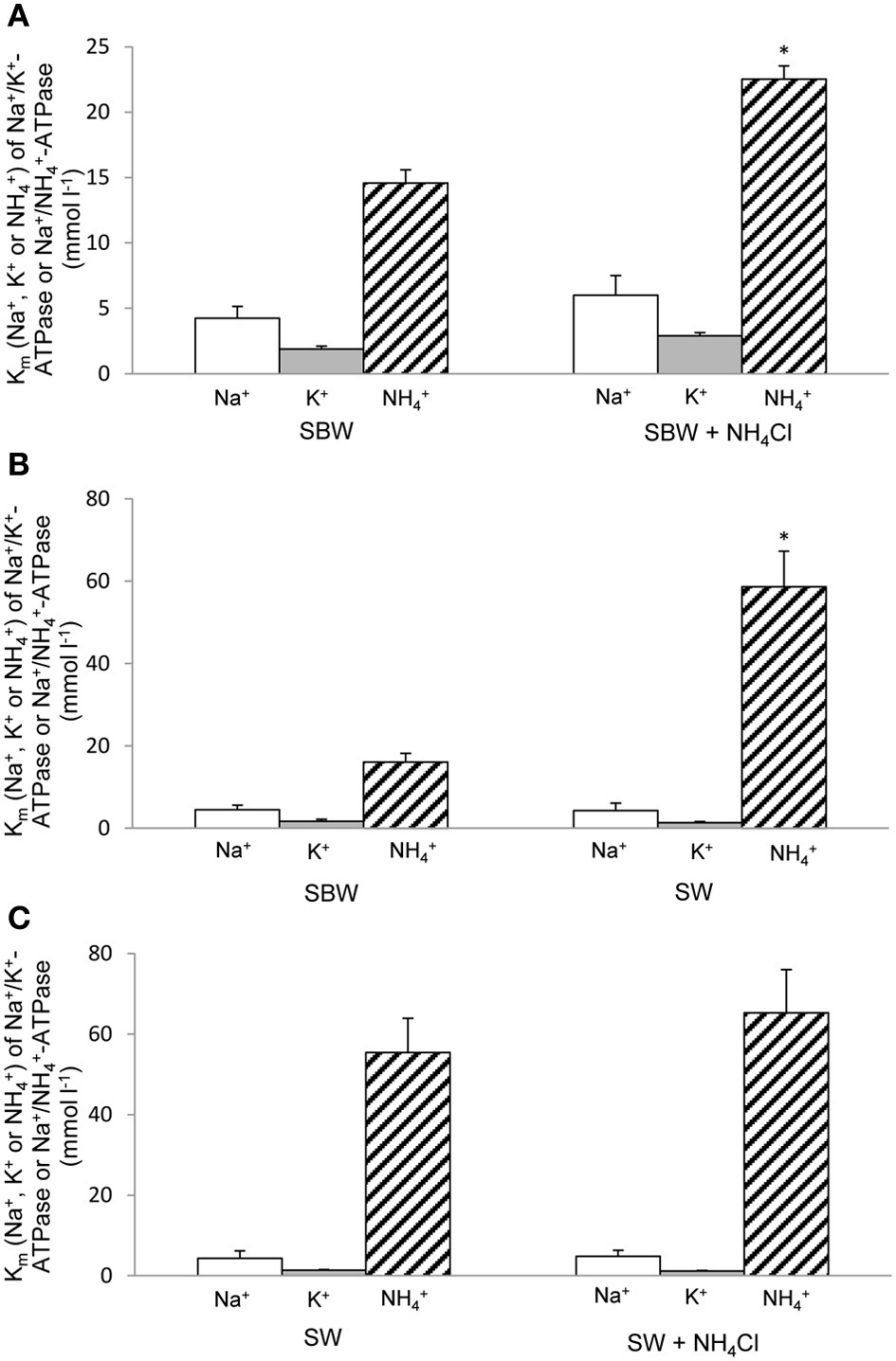
***K*_*m*_ (for Na^+^, K^+^, or NH^+^_4_) of Na^+^/K^+^(NH^+^_4_)-ATPase under various experimental conditions.** Effects of 6-days exposure to **(A)** slightly brackish water (SBW; salinity 3) containing 75 mmol l^−1^ NH_4_Cl, **(B)** seawater (SW; salinity 30), or **(C)** SW containing 50 mmol l^−1^ NH_4_Cl on *K*_*m*_ (for Na^+^, K^+^, or NH^+^_4_) of Na^+^/K^+^(NH^+^_4_)-ATPase (mmol l^−1^) from gills of *Periophthalmodon schlosseri* which had been kept in SBW for 2 weeks. Results represent mean ± s.e.m. (*N* = 4). ^*^Significant difference from the *K*_*m*_ for NH^+^_4_ of Na^+^/K^+^(NH^+^_4_)-ATPase of the corresponding control (*P* < 0.05).

**Figure 3:**
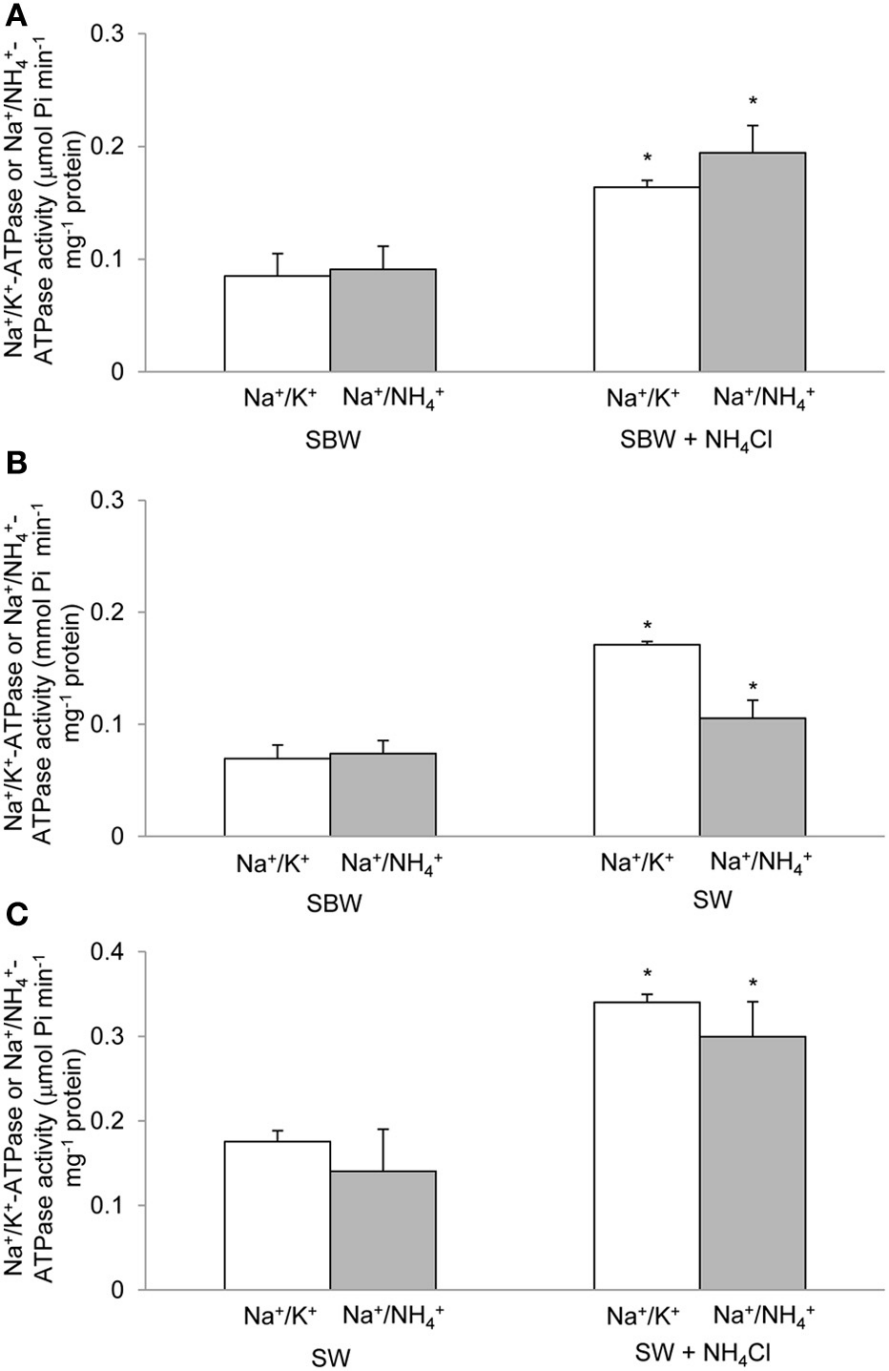
**Specific activity of Na^+^/K^+^(NH^+^_4_)-ATPase under various experimental conditions.** Effects of 6-days exposure to **(A)** slightly brackish water (SBW; salinity 3) containing 75 mmol l^−1^ NH_4_Cl, **(B)** seawater (SW; salinity 30), or **(C)** SW containing 50 mmol l^−1^ NH_4_Cl on the specific activity (μmol P_*i*_ min^−1^ mg^−1^ protein) of Na^+^/K^+^(NH^+^_4_)-ATPase from gills of *Periophthalmodon schlosseri* which had been kept in SBW for 2 weeks. Results represent mean ± s.e.m. (*N* = 4). ^*^Significant difference from the corresponding control (*P* < 0.05).

Results are presented as means ± standard errors of the mean (S.E.M.). Results presented in Figures 1–3, 10–12 were evaluated by Student *t*-test while those presented in Figures 7–9 were evaluated by One-Way analysis of variance (ANOVA), followed by multiple comparisons of means by the Tukey test, because they satisfied the criteria (i.e., normal distribution and equal variances) for parametric analyses. For data presented in Table 2, arcsin transformation was applied to achieve normal distribution, before statistical analysis by ANOVA and the Tukey test. Differences with *P* < 0.05 were regarded as statistically significant.

**Table 2:** **Effectiveness of NH^+^_4_ to activate Na^+^/K^+^-ATPase (Nka) from the gills of *Periophthalmodon schlosseri***.

**KCl or NH_4_Cl concentration (mmol l^−1^)**	Effectiveness of NH^+^_4_ to induce Nka activity (expressed as ratio of Na^+^/NH^+^_4_-ATPase activity to Na^+^/K^+^-ATPase^*^)			
	**SBW (Control)**	**75 mmol l^−1^ NH_4_Cl in SBW**	**SW**	**50 mmol l^−1^ NH_4_Cl in SW**
1	0.27 ± 0.10^a^	0.032 ± 0.006^b^	0.025 ± 0.006^b^	0.033 ± 0.014^b^
2.5	0.32 ± 0.10^c^	0.051 ± 0.006^d^	0.16 ± 0.01^cd^	0.13 ± 0.04^cd^
5	0.40 ± 0.07	0.20 ± 0.05	0.29 ± 0.03	0.36 ± 0.08
20	0.94 ± 0.05	0.64 ± 0.02	0.63 ± 0.11	0.67 ± 0.10

The ratio of Na^+^/NH^+^_4_-ATPase activity to Na^+^/K^+^-ATPase activity^*^, determined at 1, 2.5, 5, or 20 mmol l^−1^ of KCl or NH_4_Cl, from the gills of P. schlosseri pre-acclimated to slightly brackish water (SBW; salinity 3) for 2 weeks (control), or after 6-days exposure to 75 mmol l^−1^ NH_4_Cl in SBW (pH 7.0), seawater (SW; salinity 30) or 50 mmol l^−1^ NH4Cl in SW (pH 8.2). Values are means ± s.e.m. (N = 4). Comparisons are made among the different groups at each substrate concentration. Means not sharing the same letter are significantly different from each other.

* A ratio of 1.0 indicates that NH^+^_4_ had the same effectiveness as K^+^ to induce Nka activity.

## Results

### Kinetic properties of branchial Nka after exposure to various conditions

Results obtained in this study revealed that NH^+^_4_ could not effectively substitute for K^+^, especially at low substrate concentrations, to induce Nka activity from the gills of *P. schlosseri* pre-acclimated to SBW for 2 weeks (Figure 1; Table 2). For branchial Nka from these control fish in SBW, the *K*_*m*_ for K^+^ (1.6 mmol l^−1^) was substantially lower than the *K*_*m*_ for NH^+^_4_ (15 mmol l^−1^; Figure 2A), indicating that it had much greater affinity to K^+^ than NH^+^_4_. After 6-days exposure to 75 mmol l^−1^ NH_4_Cl in SBW, the *K*_*m*_ of branchial Nka for K^+^ remained relatively unchanged but the *K*_*m*_ for NH^+^_4_ increased significantly to 23 mmol l^−1^ (Figure 2A). After *P. schlosseri* was exposed to SW for 6 days, the *K*_*m*_ of branchial Nka for K^+^ also remained unchanged but the *K*_*m*_ for NH^+^_4_ increased significantly to 56 mmol l^−1^ (Figure 2B). The *K*_*m*_ for K^+^ and *K*_*m*_ for NH^+^_4_ of Nka from the gills of *P. schlosseri* exposed to 50 mmol l^−1^ NH_4_Cl in SW were comparable to those of fish exposed to SW (Figure 2C).

In general, the effectiveness of NH^+^_4_ to replace K^+^ to activate Nka (expressed as the ratio of Na^+^/NH^+^_4_-ATPase activity to Na^+^/K^+^-ATPase activity) from the gills of *P. schlosseri* was the highest at saturating substrate concentrations (0.63–0.94 at 20 mmol l^−1^; Table 2), and it decreased with decreasing substrate concentrations (0.03–0.27 at 1 mmol l^−1^; Table 2). At high concentrations (5 and 20 mmol l^−1^) of K^+^ or NH^+^_4_, exposure to 75 mmol l^−1^ NH_4_Cl in SBW, SW or 50 mmol l^−1^ NH_4_Cl in SW for 6 days had no significant effects on the effectiveness of NH^+^_4_ to substitute for K^+^ as compared to the SBW control (Table 2). However, the effectiveness of NH^+^_4_ to substitute for K^+^ to activate Nka at 1 mmol l^−1^ of K^+^ or NH^+^_4_ decreased significantly from 0.27 for the SBW control to 0.025–0.033 for fish exposed to 75 mmol l^−1^ NH_4_Cl in SBW, SW or 50 mmol l^−1^ NH_4_Cl in SW for 6 days (Table 2). Furthermore, at 2.5 mmol l^−1^ of K^+^/NH^+^_4_, the effectiveness of NH^+^_4_ to substitute for K^+^ to activate Nka from gills of fish exposed to 75 mmol l^−1^ NH_4_Cl in SBW for 6 days (0.051) was significantly lower than that of the SBW control (0.32; Table 2).

There were significant increases in activities (*V*_sat_, which was determined at saturating substrate concentrations and hence close to the theoretical *V*_max_) of Na^+^/K^+^-ATPase and Na^+^/NH^+^_4_-ATPase from the gills of *P. schlosseri* after 6-days exposure to 75 mmol l^−1^ NH_4_Cl in SBW (Figure 3A) or SW (Figure 3B) as compared to the SBW control. The *V*_sat_ of branchial Na^+^/K^+^-ATPase and Na^+^/NH^+^_4_-ATPase from fish exposed to 50 mmol l^−1^ NH_4_Cl in SW for 6 days were significantly higher than those of fish exposed to SW only (Figure 3C).

### Nucleotide sequences of branchial *nka* α isoforms and their deduced amino acid sequences

Two full cDNA sequences of *nka* α-subunits, *nka*α*1* (GenBank: KF410828) and *nka*α*3* (GenBank: KF410829), were cloned from the gills of *P. schlosseri*. As previous studies demonstrated that the set of primers used to clone and sequence *nka*α in this study could effectively “fish out” 3 *nka*α*1* isoforms, 1 *nka*α*2* isoform and 2 *nka*α*3* isoforms from the gills (Ip et al., 2012a, 2013) and brains (Chen et al., 2013; Hiong et al., 2014) of 4 other fish species, it is highly probable that the gills of *P. schlosseri* expressed only one isoform each of *nka*α*1* and *nka*α*3*.

**Figure 4:**
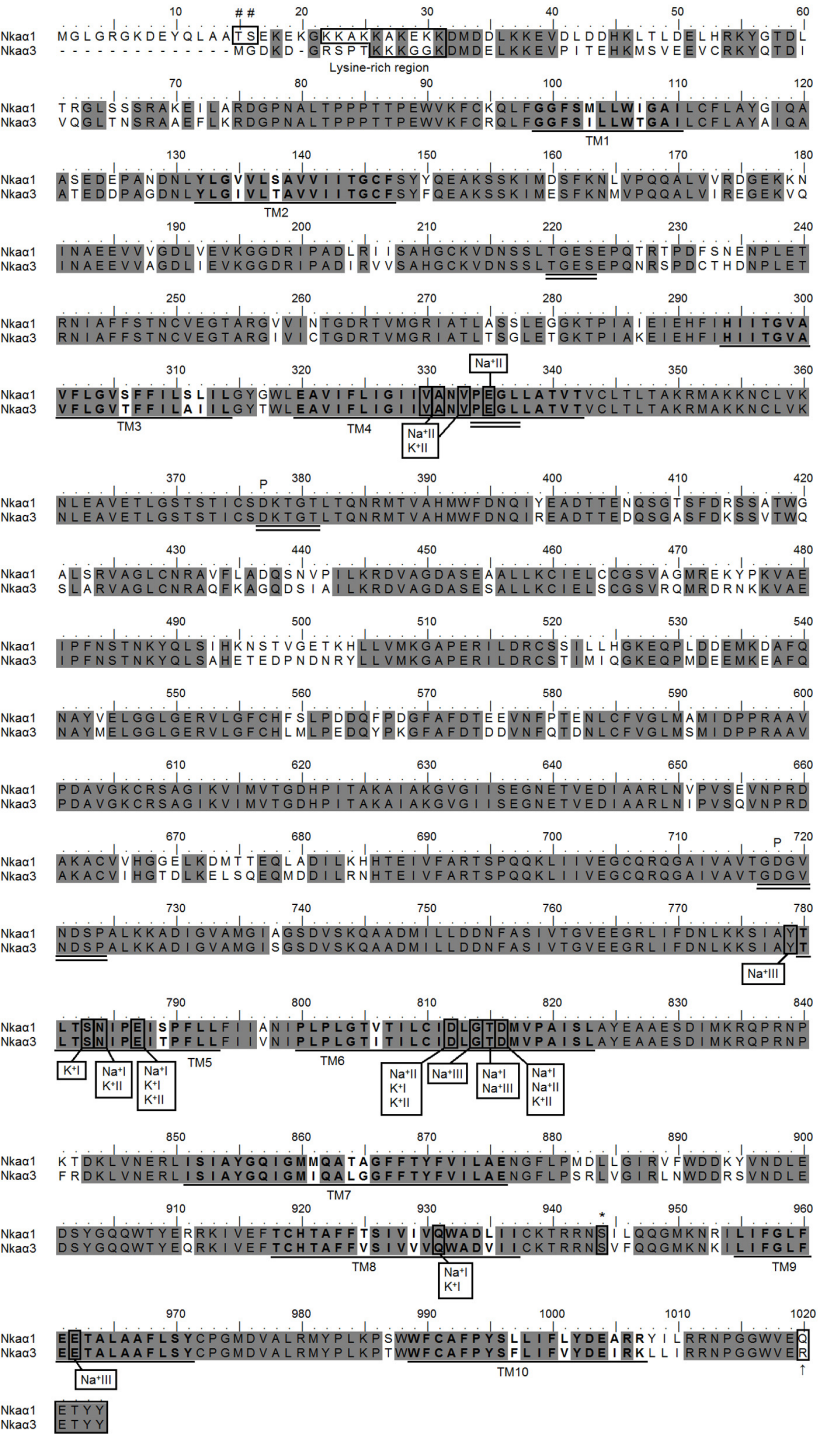
**Molecular characterization of Na^+^/K^+^-ATPase (Nka) α1 and Nkaα3.** Amino acid sequence alignment of Nkaα1 and Nkaα3 from the gills of *Periophthalmodon schlosseri*. Identical amino acid residues are indicated by shaded dark gray residues. The 10 predicted transmembrane regions (TM1-TM10) are underlined and in bold. Vertical boxes represent coordinating residues for Na^+^ or K^+^ binding. The conserved regions containing the TGES, PEGL, DKTGT, and GDGVNDSP sequence motifs are double underlined and the phosphorylation sites are indicated by a “*P*.” The asterisk and hash marks denote the amino acid residues phosphorylated by protein kinase A and protein kinase C, respectively. The lysine-rich region is indicated with a box. The KETYY motif is indicated with a box and an arrow indicating the amino acid residue that replaced arginine. The transmembrane domains were predicted using MEMSAT3 and MEMSAT-SVM provided by PSIPRED protein structure prediction server.

The coding sequence of *nka*α*1* from the gills of *P. schlosseri* contained 3075 bp, which encoded for 1024 amino acids with a calculated molecular mass of 112.5 kDa (Figure 4). In comparison, the cDNA coding sequence of *nka*α*3* was slightly shorter and contained 3030 bp, encoding 1009 amino acids with a calculated molecular mass of 111.4 kDa (Figure 4). A hydropathy analysis of Nkaα1 revealed that both *nka*α*1* and *nka*α*3* comprised 10 transmembrane domains (Figure 4). Nkaα1 and Nkaα3 from gills of *P. schlosseri* have large areas of conserved regions of Nka/NKA, which include the threonine-glycine-glutamic acid-serine (TGES) motif, the proline-glutamic acid-glycine-leucine (PEGL) motif, the aspartic acid-lysine-theronine-glycine-threonine (DKTGT) motif containing the phosphorylation site, and the glycine-aspartic acid-glycine-valine-asparagine-aspartic acid-serine-proline (GDGVNDSP) motif (Figure 4).

**Figure 5:**
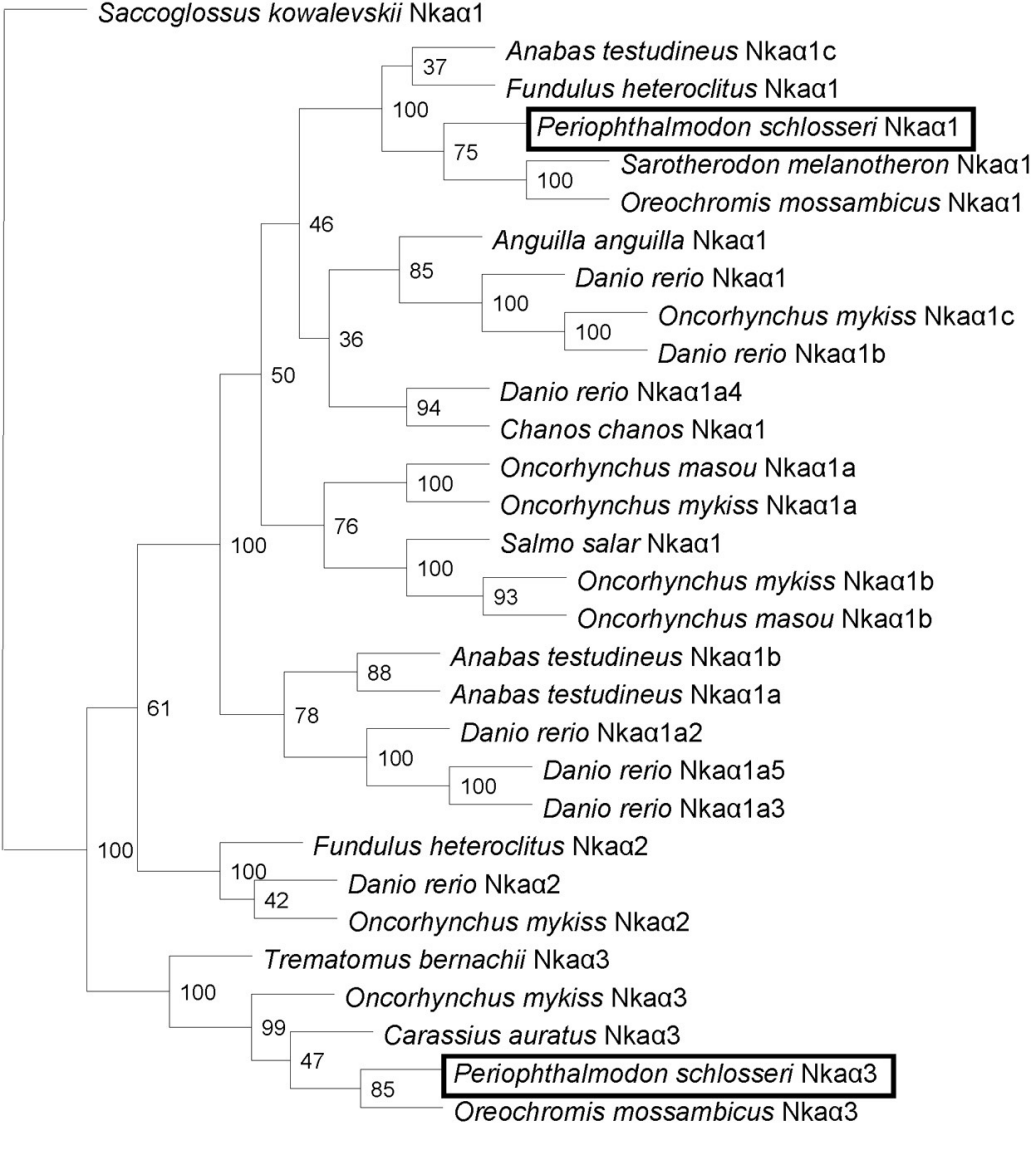
**Phenogramic analysis of Na^+^/K^+^-ATPase (Nka) α1 and Nkaα3.** A phenogram generated through phylogenetic analysis to illustrate the relationship between Nkaα1 and Nkaα3 from gills of *Periophthalmodon schlosseri* and Nkaα1, Nkaα2 and Nkaα3 of selected teleost species. Numbers presented at each branch point represent bootstrap values from 100 replicates. *Saccoglossus kowalevskii* Nka is used as an outgroup.

A comparison of Nkaα1 and Nkaα3 of *P. schlosseri* with Nka α-subunits of teleosts and NKA α-subunits from other animals revealed that they shared the highest amino acid sequence identity with teleost Nkaα1 (95.4–90.7%) and Nkaα3 (94.5–92.1%), respectively. Indeed, a phenogram of amino acid sequence similarities generated through phylogenetic analysis confirmed that the two Nkaα isoforms cloned from the gills of *P. schlosseri* were Nkaα1 and Nkaα3 (Figure 5).

**Figure 6:**
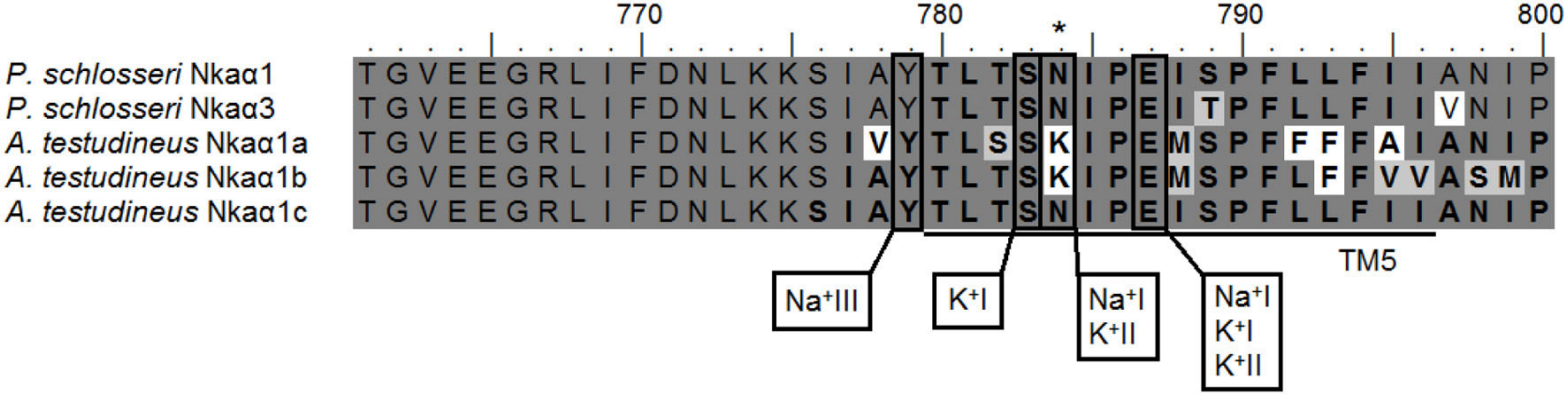
**Analysis of Na^+^/K^+^ binding site of Na^+^/K^+^-ATPase (Nka) α1 and Nkaα3.** A multiple amino acid sequence alignment of a region of Nkaα1 and Nkaα3 from gills of *Periophthalmodon schlosseri*, with Nkaα1a (GenBank: JN180940), Nkaα1b (GenBank: JN180941), and Nkaα1c (GenBank: JN180942) from the gills of *Anabas testudineus*. Identical amino acid residues are indicated by shaded black residues and similar amino acids (threshold value 60%) are indicated by shaded gray residues. Vertical boxes represent coordinating residues for Na^+^ or K^+^ binding. Asterisks indicate the amino acid residue that is similar to Nkaα1c but different from Nkaα1a and Nkaα1b.

The percentage similarities between the deduced amino acid sequence of Nkaα1 from gills of *P. schlosseri* and Nkaα1a [JN180940], Nkaα1b [JN180941], and Nkaα1c [JN180942] from gills of *A. testudineus* were 81.1, 87.7, and 93.5%, respectively; for Nkaα3 from gills of *P. schlosseri*, the respective similarities were 75.5, 78.5, and 85.0%. Hence, both Nkaα1 and Nka3 of *P. schlosseri* had the greatest similarity with Nkaα1c of *A. testudineus*. A detailed analysis of the amino acid residues constituting the K^+^ binding sites of Nkaα1 and Nkaα3 from the gills of *P. schlosseri* confirmed that they are identical to those of Nkaα1c, but different from those of Nkaα1a (freshwater-isoform) and Nkaα1b (seawater-isoform), from the gills of *A. testudineus* (Figure 6).

### mRNA expression of branchial *nka*α*1* and *nka*α*3* exposed to various conditions

**Figure 7:**
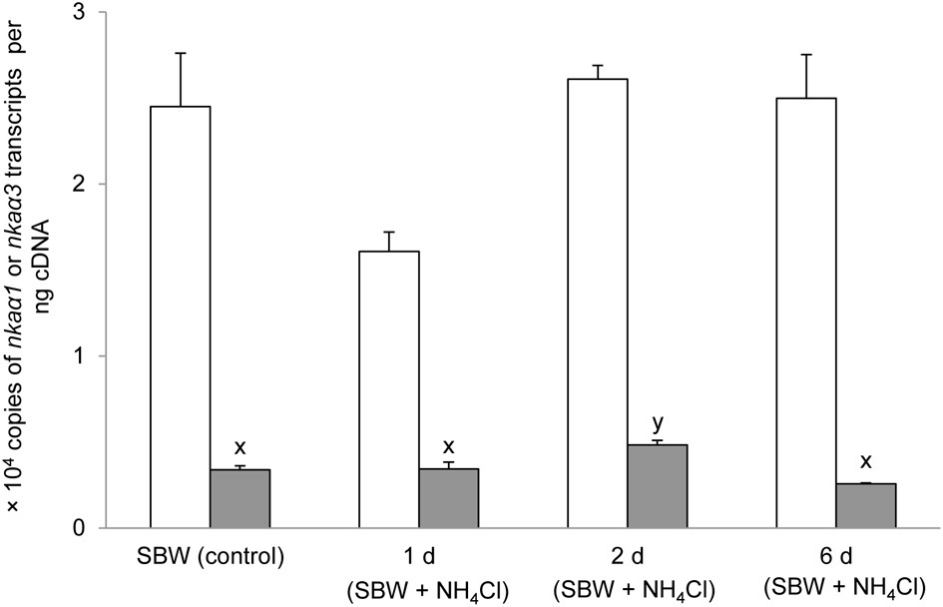
**Effects of exposure to ammonia in slightly brackish water on Na^+^/K^+^-ATPase (*nka*) α*1* and *nka*α*3* mRNA expression.** Absolute quantification (copies of transcript per ng cDNA) of *nka*α*1* (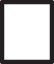) and *nka*α*3* (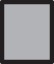) in gills of *Periophthalmodon schlosseri* kept in slightly brackish water (SBW; salinity 3) for 2 weeks (control), or after 1, 2, or 6 days of transfer from SBW to SBW containing 75 mmol l^−1^ NH_4_Cl. Results represent mean + s.e.m. (*N* = 4). Means not sharing the same letter are significantly different (*P* < 0.05).

**Figure 8:**
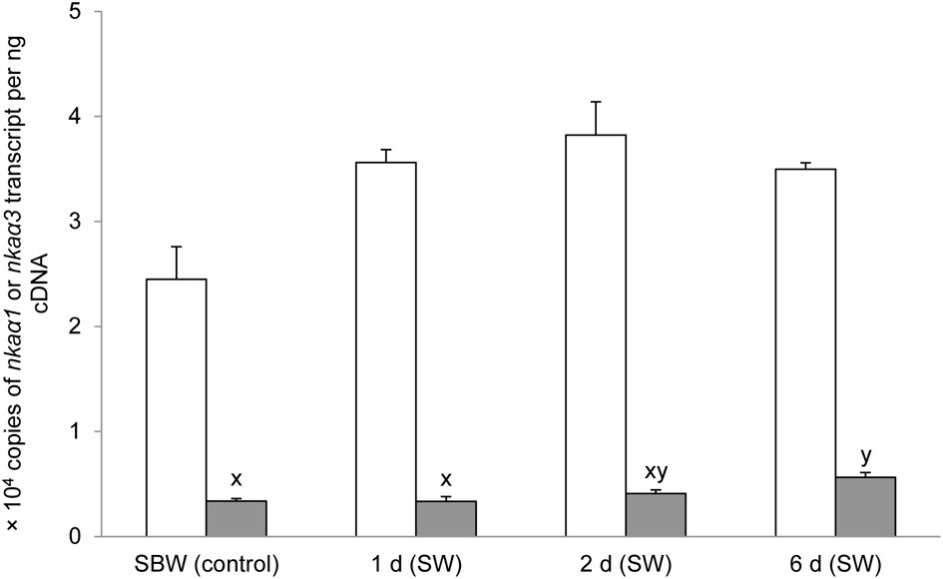
**Effects of exposure to seawater on Na^+^/K^+^-ATPase (*nka*) α*1* and *nka*α*3* mRNA expression.** Absolute quantification (copies of transcript per ng cDNA) of *nka*α*1* (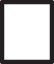) and *nka*α*3* (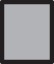) in gills of *Periophthalmodon schlosseri* kept in slightly brackish water (SBW; salinity 3) for 2 weeks (control) or after 1, 2, or 6 days of transfer from SBW to seawater (SW; salinity 30). Results represent mean + s.e.m. (*N* = 4). Means not sharing the same letter are significantly different (*P* < 0.05).

**Figure 9:**
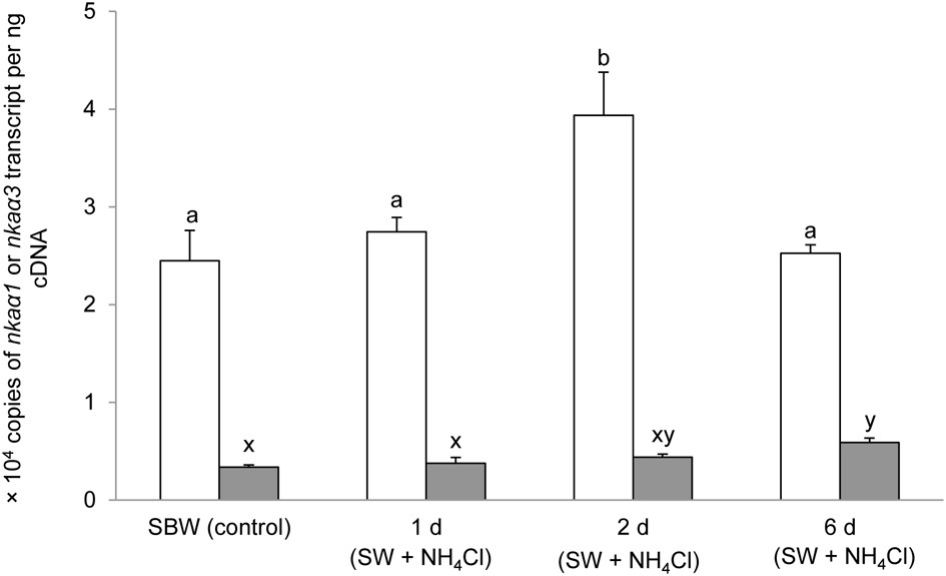
**Effects of exposure to ammonia in seawater on Na^+^/K^+^-ATPase (*nka*) α*1* and *nka*α*3* mRNA expression.** Absolute quantification (copies of transcript per ng cDNA) of *nka*α*1* (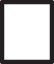) and *nka*α*3* (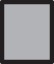) in gills of *Periophthalmodon schlosseri* kept in slightly brackish water (SBW; salinity 3) for 2 weeks (control) or after 1, 2, or 6 days of transfer from SBW to seawater (SW; salinity 30) containing 50 mmol l^−1^ NH_4_Cl. Results represent mean + s.e.m. (*N* = 4). Means not sharing the same letter are significantly different (*P* < 0.05).

Six days of exposure of *P. schlosseri* to 75 mmol l^−1^ NH_4_Cl in SBW had no significant effects on the mRNA expression of *nka*α*1* in the gills; however, there was a transient but significant increase in the branchial mRNA expression of *nka*α*3* on day 2 (Figure 7). Similarly, 6-days exposure to SW had no significant effects on the mRNA expression of *nka*α*1* in the gills of *P. schlosseri*, but there was a significant increase in the branchial mRNA expression of *nka*α*3* on day 6 (Figure 8). However, upon exposure to 50 mmol l^−1^ NH_4_Cl in SW, there were significant increases in the mRNA expression of *nka*α*1* and *nka*α*3* (Figure 9) in the gills of *P. schlosseri* on day 2 and 6, respectively.

### Protein abundance of branchial *nka*α exposed to various conditions

**Figure 10:**
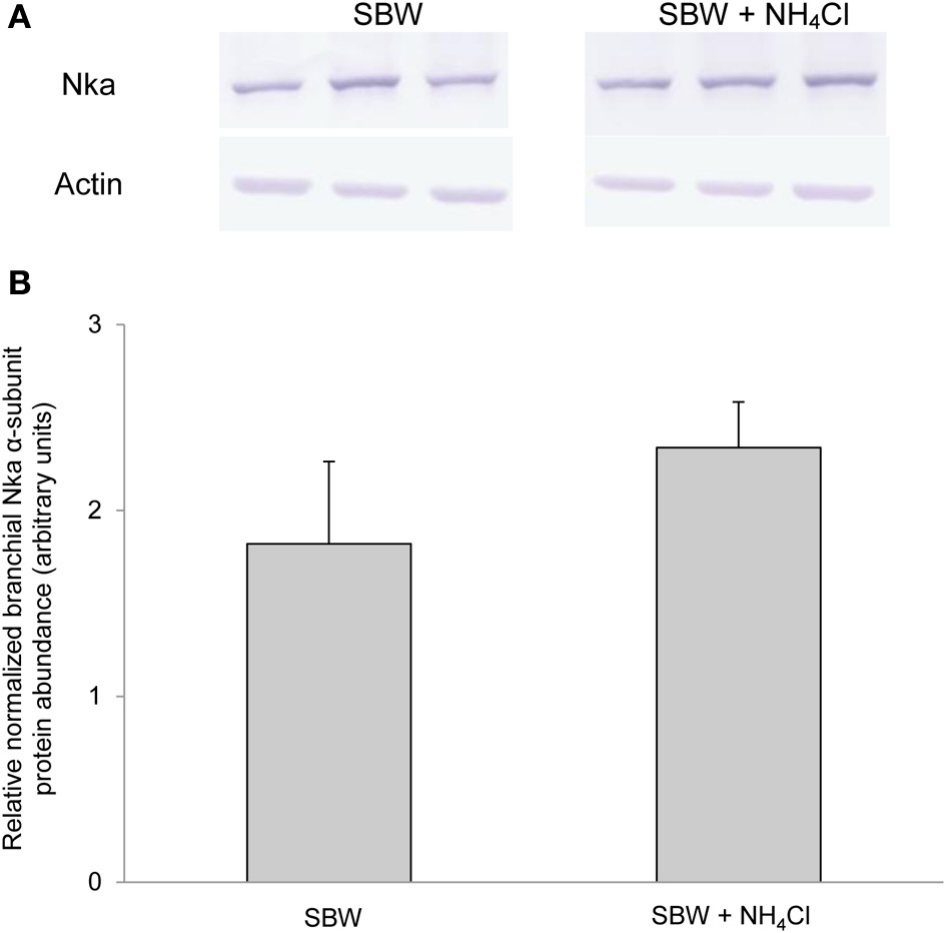
**Effects of exposure to ammonia in slightly brackish water on Na^+^/K^+^-ATPase (Nka) protein abundance.** Protein abundance of Nka based on the α5 anti-NKA antibody, which is known to react with all Nka/NKA α-isoforms, in gills of *Periophthalmodon schlosseri* kept in slightly brackish water (SBW; salinity 3) for 2 weeks (control) or after 6 days of transfer from SBW to SBW containing 75 mmol l^−1^ NH_4_Cl. **(A)** The immunoblots of Nka and actin. **(B)** The intensity of the Nka band was normalized with respect to that of actin. Results represent mean + s.e.m. (*N* = 3).

**Figure 11:**
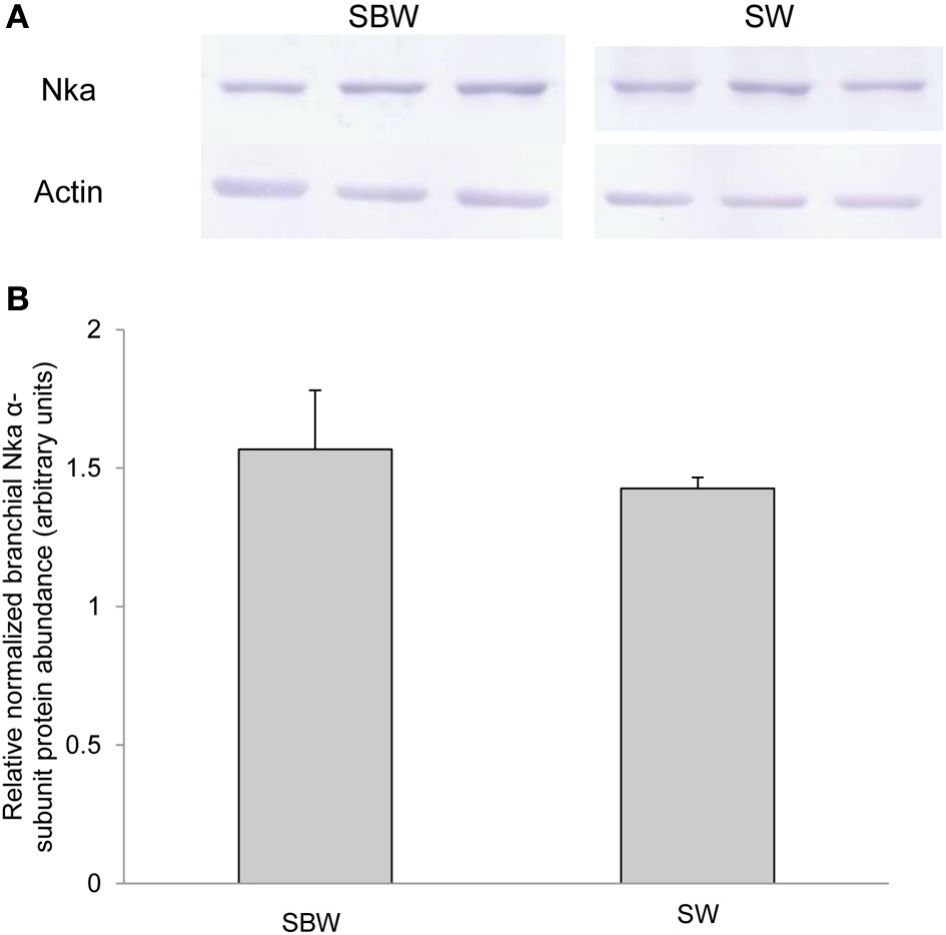
**Effects of exposure to seawater on Na^+^/K^+^-ATPase (Nka) protein abundance.** Protein abundance of Nka based on the α5 anti-NKA antibody which is known to react with all Nka/NKA α-isoforms of gills of *Periophthalmodon schlosseri* kept in slightly brackish water (SBW; salinity 3) for 2 weeks (control) or after 6 days of transfer from SBW to seawater (SW; salinity 30). **(A)** The immunoblots of Nka and actin. **(B)** The intensity of the Nka band was normalized with respect to that of actin. Results represent mean + s.e.m. (*N* = 3).

**Figure 12:**
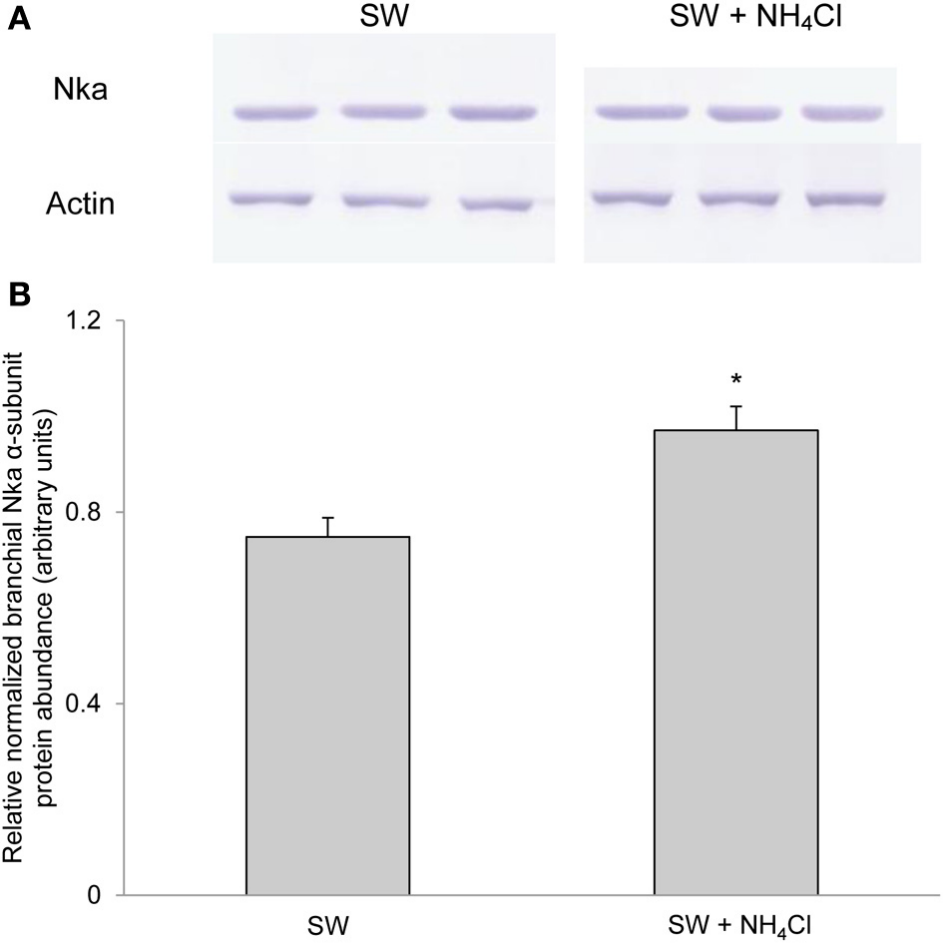
**Effects of exposure to ammonia in seawater on Na^+^/K^+^-ATPase (Nka) protein abundance.** Protein abundance of Nka based on the α5 anti-NKA antibody which is known to react with all Nka/NKA α-isoforms of gills of *Periophthalmodon schlosseri* after 6 days of transfer from slightly brackish water (SBW; salinity 3) to seawater (SW; salinity 30; control) or after 6 days of transfer from SBW to SW containing 50 mmol l^−1^ NH_4_Cl. **(A)** The immunoblots of Nka and actin. **(B)** The intensity of the Nka band was normalized with respect to that of actin. Results represent mean + s.e.m. (*N* = 3). ^*^Significantly different from the control (*P* < 0.05).

Based on Western blotting results using the α5 anti-NKA antibody, which reacted comprehensively with fish Nka α-subunit isoforms, the protein abundance of Nkaα remained unchanged in the gills of *P. schlosseri* after 6-days exposure to 75 mmol l^−1^ NH_4_Cl in SBW (Figure 10) or SW (Figure 11) as compared with that of the control pre-acclimated to SBW for 2 weeks. By contrast, 6-days exposure to 50 mmol l^−1^ NH_4_Cl in SW led to a slight but significant increase in the protein abundance of Nkaα in the gills of *P. schlosseri* (Figure 12).

## Discussion

### K_*m*_ for NH^+^_4_ is substantially greater than K_*m*_ for K^+^ under all experimental conditions

*Periophthalmodon schlosseri* could survive abrupt transfer between SBW and SBW containing 75 mmol l^−1^ NH_4_Cl and between SBW and SW with or without 50 mmol l^−1^ NH_4_Cl without mortality. Exposure of *P. schlosseri* to SW or to ammonia in SBW or SW resulted in significant increases in the *V*_sat_ of branchial Nka, with Na^+^ and K^+^/NH^+^_4_ as substrates, indicating that branchial Nka was essential for salt excretion in a hyperosmotic medium and active NH^+^_4_ excretion in a medium containing high concentrations of ammonia.

For branchial Nka of *P. schlosseri* pre-acclimated to SBW for 2 weeks, the *K*_*m*_ for K^+^ (1.6 mmol l^−1^) was substantially lower than the *K*_*m*_ for NH^+^_4_ (15 mmol l^−1^), indicating that the K^+^ binding site had high affinity for K^+^ and low affinity for NH^+^_4_. Taking the plasma K^+^ and NH^+^_4_ concentrations as 10 mmol l^−1^ and 1 mmol l^−1^, respectively, it would mean that branchial Nka would be saturated with K^+^, and therefore it is highly improbable that branchial Nka would transport NH^+^_4_ in replacement of K^+^. More importantly, after 6-days exposure to 75 mmol l^−1^ NH_4_Cl in SBW, the *K*_*m*_ for NH^+^_4_ increased significantly to 23 mmol l^−1^ with the *K*_*m*_ for K^+^ remained relatively unchanged. These results indicate that branchial Nka could even better differentiate NH^+^_4_ from K^+^ when *P. schlosseri* was challenged with environmental ammonia. Hence, it can be concluded that the main function of Nka in active NH^+^_4_ excretion is to maintain intracellular Na^+^ and K^+^ homeostasis, and NH^+^_4_ is not directly transported into ionocytes through the basolateral Nka during active NH^+^_4_ excretion as proposed previously for *P. schlosseri* (Randall et al., 1999; Weihrauch et al., 2009; Hwang et al., 2011).

For *P. schlosseri* exposed to SW, the *K*_*m*_ of the branchial Nka for K^+^ remained unchanged while the *K*_*m*_ for NH^+^_4_ increased significantly to 56 mmol l^−1^. Thus, SW exposure apparently led to increases in expression and/or activity of a Nkaα isoform which had even lower affinity for NH^+^_4_ as compared with the control fish. These results have important bearing on the behavior and physiology of *P. schlosseri*, which builds burrows in mudflats. During high tides, *P. schlosseri* seeks refuge into mud burrows, which may contain water with high salt and ammonia concentrations. During the breeding season, eggs are laid inside the burrows, and ammonia produced during embryonic development could further increase the ammonia concentration therein. Therefore, it would be advantages for *P. schlosseri* to readily increase the selectivity of its branchial Nka for K^+^ over NH^+^_4_ during SW exposure. Overall, these results indicate that all the Nkaα isoforms expressed in the gills of *P. schlosseri* in SBW, SW, or ammonia in SBW/SW would have K^+^ binding sites that could effectively differentiate K^+^ from NH^+^_4_. Thus, we proceeded to clone *nka*α isoforms from the gills of *P. schlosseri* in order to characterize the K^+^ binding sites of their deduced amino acid sequences.

### Molecular characterization of *nka*α isoforms

Two *nka*α isoforms, *nka*α*1* and *nka*α*3*, were cloned from the gills of *P. schlosseri*. Similarly, Feng et al. (2002) reported the expression of two *nka* isoforms, *nka*α*1* and *nka*α*3* in the gills of *O. mossambicus*. Using isoform specific anti-Nkaα antibodies, Lee et al. (1998) demonstrated that Nkaα1 and Nkaα3 were present in branchial ionocytes of *O. mossambicus*, and showed that their expressions were responsive to salinity changes.

Three Na^+^ and two K^+^ binding sites are known to be present in the NKA α-subunit (Ogawa and Toyoshima, 2002). The coordinating residues present in the binding sites are arranged within the transmembrane domains such that the release of one type of cation coordinates with the binding of the other. Based on the homology modeling of human NKAα (Ogawa and Toyoshima, 2002), these coordinating residues were conserved in Nkaα1 and Nkaα3 from the gills of *P. schlosseri*.

Morth et al. (2007) reported a 96% reduction in Na^+^ affinity when five amino acid residues (KETYY) were deleted from the C-terminus of NKA. Although the KETYY motif was present in both Nka α-subunit isoforms of *P. schlosseri*, the lysine residue was replaced by glutamine and arginine in Nkaα1 and Nkaα3, respectively. This could indicate that the Na^+^ affinity of Nkaα1 could be different from that of Nkaα3. Mutations studies have shown that Asparagine-786 is critical for both Na^+^ and K^+^ binding (Pedersen et al. 1998). For *A. testudineus*, there was a replacement of asparagine by lysine in position 786 of Nkaα1a (freshwater-isoform) and Nkaα1b (seawater-isoform) (Ip et al., 2012a). However, similar to Nkaα1c (ammonia-isoform) of *A. testudineus* (Ip et al., 2012a), no replacement of the equivalent Asparagine-784 was found in both Nkaα1 and Nkaα3 of *P. schlosseri*.

The amino acid residues constituting the K^+^ binding sites of Nkaα1 and Nkaα3 from the gills of *P. schlosseri* were identical to those of Nkaα1c, but different from those of Nkaα1a and Nkaα1b, from the gills of *A. testudineus* (Ip et al., 2012a). Ip et al. (2012a) demonstrated that exposure of *A. testudineus* to ammonia led to significant increases in the mRNA expression of *nka*α*1c*, the overall Nkaα protein abundance and the activity of Nka in its gills. Furthermore, there were increases in the *K*_*m*_ of Nka for K^+^ and NH^+^_4_, with the increase in *K*_*m*_ for NH^+^_4_ being much greater than that for K^+^, indicating a decrease in the effectiveness of NH^+^_4_ to replace K^+^ to activate branchial Nka in *A. testudineus* exposed to ammonia. Thus, Ip et al. (2012a) concluded that the up-regulation of *nka*α*1c* expression served to maintain intracellular Na^+^ and K^+^ homeostasis by removing excess Na^+^ from, and transporting K^+^ in preference to NH^+^_4_ into, ionocytes. The similarity in the K^+^ binding sites between Nkaα1/Nkaα3 of *P. schlosseri* and Nkaα1c of *A. testudineus* corroborate the fact that the overall Nka activity from the gills of *P. schlosseri* exhibited high substrate specificity for K^+^, that is, low *K*_*m*_ for K^+^ and high *K*_*m*_ for NH^+^_4_.

Incidentally, basolateral Nka is also known to be involved in branchial ammonia excretion in aquatic crustaceans (Towle and Holleland, 1987; Towle et al., 2001). Masui et al. (2002, 2009) demonstrated that the branchial Nka activity from the marine blue crab, *Callinectes danae*, was synergistically stimulated by NH^+^_4_ and K^+^. Upon the addition of NH^+^_4_ to the assay medium containing an optimal concentration of K^+^, the activity of Nka increased by ~90%. It was speculated that K^+^ and NH^+^_4_ were bound to different sites of Nka in *C. danae* (Masui et al., 2002, 2009). Furriel et al. (2004) reported a similar phenomenon for the branchial Nka from the freshwater shrimp, *Macrobrachium olfersii*. They proposed that at high NH^+^_4_ concentrations, a new binding site for NH^+^_4_ was exposed in the Nka from *M. olfersii*. This new binding site modulated the Nka activity independently of K^+^ after binding to NH^+^_4_ (Furriel et al., 2004). However, to date, no molecular or structural information is available on the substrate binding sites of Nka from the gills of *M. olfersii*. Notably, we were unable to demonstrate a synergistic effect of K^+^ and NH^+^_4_ in inducing the activity of Nka from the gills of *P. schlosseri* (Chew and Ip, unpublished results). In addition, molecular characterization of Nkaα1 and Nkaα3 from the gills of *P. schlosseri* exposed to ammonia in SBW revealed, as usual, the presence of only two binding sites for K^+^ and three binding sites for Na^+^.

It is probable that Nka was regulated by phosphorylation/dephosphorylation in the gills of *P. schlosseri* exposed to various environmental conditions. Both cAMP-dependent protein kinase A (PKA) and protein kinase C (PKC) are known to be involved in the phosphorylation of the NKA α-subunit (Aperia et al., 1994). One possible site of cAMP-dependent PKA phosphorylation is the Serine-944 of the NKA α-subunit from kidneys of the rat and the giant toad, *Bufo marinus* (Beguin et al., 1994; Feschenko and Sweadner, 1994). This site was conserved in Nkaα1 and Nkaα3 of *P. schlosseri*. Beguin et al. (1994) identified Threonine-10 and Serine-11 as cAMP-dependent PKC phosphorylation sites in the NKA of *B. marinus* by site-directed mutagenesis. For *P. schlosseri*, these two PKC phosphorylation sites corresponded to Threonine-15 and Serine-16 in Nkaα1, but were absent from Nkaα3. Feschenko and Sweadner (1995) identified two cAMP-dependent PKC phosphorylation sites, Serine-11 and Serine-18, with different phosphorylatability in rat kidney NKAα1. For *P. schlosseri*, only Serine-16 was found in Nkaα1, but both serine residues were absent from Nkaα3. These differences in phosphorylation sites between Nkaα1 and Nkaα3 might have important bearing on the interpretation of the changes in mRNA expression of *nka*α*1* and *nka*α*3* and the protein abundance of Nkaα in the gills of *P. schlosseri* in response to various experimental conditions.

### Exposure to ammonia in SBW

Exposure of *P. schlosseri* to 75 mmol l^−1^ NH_4_Cl in SBW led to increases in Nka activity and mRNA expression of *nka*α*3*, but not the overall Nkaα protein abundance in its gills. There was a significant increase (~2-fold) in the *V*_sat_ of branchial Na^+^/K^+^-ATPase and Na^+^/NH^+^_4_-ATPase. This corroborates the proposition that there could be an increase in the entry of Na^+^, NH^+^_4_, and 2Cl^−^ into the ionocyte through the basolateral Nkcc1 during active ammonia excretion, and an increase in Nka activity was needed to pump the excess Na^+^ back to the blood. As such, ouabain would predictably have an inhibitory effect on active NH^+^_4_ excretion, as reported by Randall et al. (1999), not because of the direct involvement of Nka in NH^+^_4_ transport, but because a decrease in Nka activity would necessarily result in a reduction in the efficiency of Nkcc1 to transport Na^+^ together with K^+^/NH^+^_4_ and 2Cl^−^ down the electrochemical gradient of Na^+^ generated by Nka.

Increases in Nka activity in fish gills can be due to an up-regulation of *nka*α mRNA expression leading to an increase in Nkaα protein abundance (Singer et al., 2002; Tipsmark et al., 2002; Scott et al., 2004; Lin et al., 2006) and/or a modulation of the enzyme's hydrolytic rate (Crombie et al., 1996; Bystriansky et al., 2007). In addition, Nka activity can be rapidly modulated by several protein kinases that directly phosphorylate/dephosphorylate Nka and/or proteins involved in the trafficking of Nka between plasma membrane and intracellular endosomal pools (Blanco and Mercer, 1998). For instance, PKA (Tipsmark and Madsen, 2001) and PKC (Crombie et al., 1996) have been shown to alter branchial Nka activities in the brown trout (*Salmo trutta*) and the Atlantic cod (*Gadus morhua*), respectively. In the case of *P. schlosseri* exposed to ammonia in SBW, there was no significant change in the comprehensive Nkaα protein abundance after 6-days ammonia exposure despite a significant increase in the mRNA expression of *nka*α*3* in the gills on day 2 of exposure to ammonia. Based on the quantities of *nka*α*1* (2.4 × 10^4^ copies) and *nka*α*3* (0.35 × 10^4^ copies) transcripts expressed in the gills of *P. schlosseri* pre-acclimated to SBW, it was apparent that *nka*α*1* was the predominate isoform. Hence, it is not unexpected that the transient increase in mRNA expression of *nka*α*3* did not result in a significant increase in the total protein abundance of Nkaα.

Overall, these results suggest that the ammonia-induced increase in Nka activity in the gills of *P. schlosseri* could be attributed mainly to post-translational modification. This could be of physiological significance to *P. schlosseri* which is frequently found moving on land during low tide and hiding in a burrow during high tide. Amino acids serve as a major energy source for its locomotor activity on land (Ip et al., 2001b), and the catabolism of these amino acids would result in an increase in the production of ammonia which is actively excreted into fenestrae of the gills formed by intrafilamentous interlamellar fusions (Chew et al., 2007). On the other hand, *P. schlosseri* could be exposed to high concentrations of ammonia when it is inside the burrow, and needs to actively excrete the ammonia therein. As there are two tides daily in the tropics and each low/high tide lasts approximately 6 h, *P. schlosseri* must have the ability to adapt promptly to environmental changes. It would be inefficient if *P. schlosseri* was to modulate its branchial Nka activity predominantly through transcriptional and/or translational changes which are relatively slow processes as compared to resorting to post-translational modification.

### Exposure to SW

It has been established that SW acclimation induces an increase in the Nka activity from the gills of many fish species (see Hwang and Lee, 2007 for a review). Indeed, 6-days SW acclimation led to a significant increase in the *V*_sat_ of branchial Na^+^/K^+^-ATPase and Na^+^/NH^+^_4_-ATPase in *P. schlosseri*. Since exposure of *P. schlosseri* to SW also resulted in a significant increase in the mRNA expression and protein abundance of *nkcc1a*/Nkcc1a in its gills (Chew and Ip, unpublished results), it is probable that NaCl extrusion through the gills of *P. schlosseri* exposed to SW is mediated by the basolateral cotransport of Na^+^, K^+^, and 2Cl^−^ through Nkcc1a, coupled with the apical exit of Cl^−^ through the apical cystic fibrosis transmembrane conductance regulator (Cftr) and the paracellular extrusion of Na^+^ (see Hwang and Lee, 2007; Evans, 2008 for reviews). Being a euryhaline brackish water fish, how *P. schlosseri* regulate the paracellular movement of Na^+^ instantaneously after abrupt transfer between SW and SBW is unclear at present. Although the mRNA expression of *nka*α*3* increased significantly in the gills of *P. schlosseri* exposed to SW for 6 days, the comprehensive Nkaα protein abundance remained unchanged. Therefore, similar to exposure to ammonia in SBW, the increase in Nka activity during SW exposure could be attributed mainly to post-translational modification.

In rainbow trout (*O. mykiss*), Atlantic salmon (*S. salar*), and Arctic char (*Salvelinus alpinus*), SW acclimation involves the differential regulation of *nkaα1a* and *nkaα1b*, which are freshwater- and seawater-isoforms, respectively (Richards et al., 2003; Bystriansky et al., 2006, 2007). Similarly, Ip et al. (2012a) obtained results which suggested *nkaα1a* and *nkaα1b* as the freshwater- and seawater-isoforms, respectively, in the gills of *A. testudineus*, which can progressively acclimate from fresh water to SW. In euryhaline freshwater fishes, changes in ionocyte types are critical to branchial osmoregulatory acclimation (Hiroi et al., 1999). For *A. testudineus*, the small Nkaα1a-positive ionocytes are replaced by the large Nkaα1b-positive ionocytes during a progressive acclimation from fresh water to SW, and the replacement process involves extensive apoptosis in the gills (Ching et al., 2013). By contrast, our results indicated the absence of distinct freshwater- and seawater-Nkaα isoforms in the gills of *P. schlosseri*. It is probable that the lack of delineation between freshwater- and seawater-Nkaα isoforms and demarcation between freshwater- and seawater-ionocytes are pre-requisites for euryhaline brackish water fishes like *P. schlosseri* to survive large and abrupt changes in salinity.

### Exposure to ammonia in SW

Exposure of *P. schlosseri* to 50 mmol l^−1^ NH_4_Cl in SW resulted in increases in Nka activity, mRNA expression of *nka*α*1* and *nka*α*3* and the overall Nkaα protein abundance in its gills. Ammonia toxicity in fishes increases in SW because of multiple reasons. SW has a pH of 8.2, at which the ratio of NH_3_ to NH^+^_4_ would be greater than that at pH 7.0 in SBW (or fresh water), and NH_3_ is the predominant species that permeate biomembranes. More importantly, fishes dehydrate in SW and therefore need to imbibe SW and excrete excess salt through the gills. Ingestion of SW containing ammonia would lead to a large influx of ammonia through the digestive tract in addition to the branchial and cutaneous epithelial surfaces, and the formation of paracellular route for the extrusion of excess Na^+^ through the branchial epithelium may also result in increased influx of ammonia. Hence, the ability of *P. schlosseri* to survive in 50 mmol l^−1^ NH_4_Cl in SW at pH 8.0 is extraordinary. Indeed, exposure of *P. schlosseri* to ammonia in SW led to a further increase (~2-fold) in *V*_sat_ of branchial Na^+^/K^+^-ATPase and Na^+^/NH^+^_4_-ATPase as compared with exposure to SW only. Notably, the *V*_sat_ of 0.35 μmol Pi min^−1^ mg^−1^ protein exhibited by the gills of *P. schlosseri* exposed to ammonia in SW could be the highest activity recorded for fish gills. The increase in *V*_max_ was accompanied with significant increases in mRNA expression of *nka*α*1* on day 2 and *nka*α*3* on day 6, which together contributed to a moderate increase in the overall protein abundance of Nkaα, indicating that, once again, post-translational modification could be an important modulating factor. At present, it is uncertain whether active salt excretion and active NH^+^_4_ excretion involved different types of ionocytes in the gills of *P. schlosseri* exposed to ammonia in SW.

### Perspective

Ip et al. (2012a,b) reported that active excretion of NH^+^_4_ through the gills of *A. testudineus* in fresh water involved a type of Nka-immunopositive ionocytes with apical Cftr and basolateral Nkcc distinctly smaller than those Nka-immunopositive ionocytes involved in SW acclimation. They suggested that the active excretion of NH^+^_4_ through some unknown apical NH^+^_4_ channels could be driven by the transmembrane electrical potential generated by the efflux of Cl^−^/HCO^−^_3_ through the apical Cftr. However, it is highly probable that the mechanism of active ammonia excretion in *P. schlosseri* would be different from that in *A. testudineus* because *P. schlosseri* acidifies the branchial water and the external medium during active NH^+^_4_ excretion (Ip et al., 2004b), but *A. testudineus* alkalinizes the branchial water instead (Ip et al., 2012b). Since *P. schlosseri* can survive abrupt transfer between SBW and SW with or without high concentrations of ammonia, it is probable that its gills comprise multiple types of ionocytes for hyperionic regulation in SBW, hypoosmotic hypoionic regulation in SW, and active NH^+^_4_ excretion in high concentrations of environmental ammonia. Hence, it would be rewarding in the future to examine the types of ionocytes present in the gills of *P. schlosseri*, with emphases on their association with a specific Nkaα isoform and other relevant ion transporters (e.g., Nkcc1, Cftr, H^+^-ATPase, Na^+^/H^+^ exchanger and Rhesus glycoproteins), and to elucidate how they can be functionally switched on and off instantly during abrupt changes in salinity (e.g., between SBW and SW) and abrupt exposure to environmental ammonia.

### Conflict of interest statement

The authors declare that the research was conducted in the absence of any commercial or financial relationships that could be construed as a potential conflict of interest.
